# Matrisome remodeling in the myocardium of hypertrophic cardiomyopathy; novel targets for molecular diagnostics

**DOI:** 10.3389/fcell.2025.1641584

**Published:** 2025-08-06

**Authors:** Ayman M. Ibrahim, Hasnaa A. Elfawy, Cesare M. Terracciano, Magdi Yacoub

**Affiliations:** ^1^ Department of Zoology, Faculty of Science, Cairo University, Giza, Egypt; ^2^ Institute of Cardiovascular Physiology, University Medical Center Göttingen, Georg-August, University Göttingen, Göttingen, Germany; ^3^ Aswan Heart Center, Magdi Yacoub Heart Foundation, Aswan, Egypt; ^4^ National Heart and Lung Institute, Imperial College London, London, United Kingdom

**Keywords:** HCM, ECM, matrisome, inflammation, secretome

## Abstract

Hypertrophic cardiomyopathy (HCM) is an inherited cardiac disorder characterized by left ventricular thickening and extracellular matrix (ECM) remodeling, often manifested as increased interstitial fibrosis that impair muscle function. The clinical and pathological presentations, as well as the genetic background, vary among patients, making HCM a heterogeneous disease with diverse clinical phenotyping and responses to treatment. In HCM, the myocardium exhibits an increased secretion of inflammatory mediators and ECM proteins, indicating a stress response to myocardial pathogenesis. The production of these ECM proteins is regulated by the interaction between cardiomyocytes and the surrounding stroma, including cardiac fibroblasts, immune cells, and microvasculature. This crosstalk defines the responsiveness to injury and the progression of the disease. In this review, we aim to dissect the composition of myocardial ECM in relation to HCM development, highlighting the key cellular contributions to ECM remodeling and identifying potential molecular targets for personalized diagnostics and therapeutics.

## 1 Introduction

A distinctive hallmark of Hypertrophic Cardiomyopathy (HCM) is the thickening of the left ventricle accompanied by ECM remodeling, typically characterized by increased interstitial fibrosis (Maron, 2002; Marian and Braunwald, 2017). The disease exhibits heterogeneity in clinical and pathological presentations, as well as in genetic backgrounds, resulting in diverse responses to treatment (Marian and Braunwald, 2017; Coats et al., 2018). This heterogeneity can be attributed to the highly variable genetic and epigenetic etiology that triggers pathological mechanisms extending beyond the sarcomere, and further beyond the myocardium (Chou and Chin, 2021; Repetti et al., 2021). The ECM constitutes an intricate network of proteins essential for preserving the structural integrity and functional homeostasis of cardiac tissue under various stimuli in both physiological and pathological conditions (Hynes and Naba, 2012; Naba et al., 2012). This dynamic entity continuously undergoes remodeling to adapt to the changing demands of the heart in health and disease (Rienks et al., 2014). Maintaining a balance in collagen synthesis and degradation, protease activity, and the presence of fibulins, cytokines, and chemokines is critical for preserving cardiac function (Rienks et al., 2014; Fan et al., 2012). Therefore, comprehensive understanding of various interactions among these ECM components in physiological and pathological states is imperative for the development of targeted therapies aimed at mitigating adverse cardiac remodeling.

**FIGURE 1:**
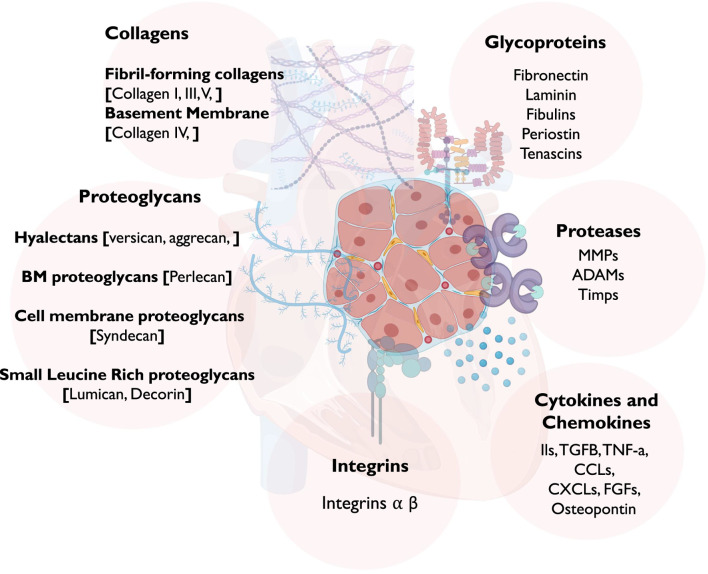
A schematic diagram showing the different components of the ECM in the myocardium. Abbreviations: MMP, Matrix Metalloproteases; ADAM, disintegrin and metalloproteinase; TGF-β, transforming growth factor beta; TNF, tumor necrosis factor; CCL, chemokine ligands; CXCL, CXC subfamily of chemokines; and FGF, fibroblast growth factor.

**FIGURE 2:**
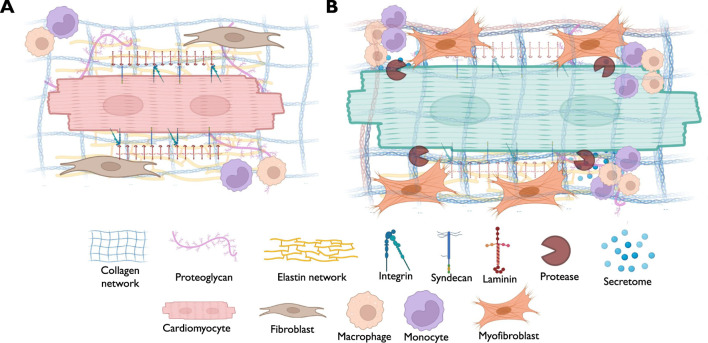
A schematic diagram demonstrating the possible alterations in different components of myocardial ECM in association with muscle hypertrophy. **(A)** The scheme points out to the normal status of the myocardium where cardiomyocytes are physiological interacting with the neighboring cells such as cardiac fibroblasts and immune cells, with intact basement membrane and receptors landscape. **(B)** The scheme highlights the pathological alterations associated with cardiomyocytes hypertrophy, such as the activation of cardiac fibroblasts that drive ECM deposition and turnover, along with the infiltration of immune cells, inducing inflammatory response.

Currently, there are approximately 300 proteins recognized as ECM proteins (Matrisome) (Hynes and Naba, 2012), comprising collagens, proteoglycans, elastin, and glycoproteins (Figure 1). Each of these proteins possesses unique physical and biochemical characteristics, and their distribution is controlled via factors contributing to ECM remodeling, such as proteases, and molecules facilitating cell-ECM interaction, such as integrins, syndecans, and other receptors (Rienks et al., 2014; Fan et al., 2012). Maintaining the balance of these proteins within myocardial tissue is essential for responding to both physiological and pathological signals [Figure 2].

## 2 Myocardial ECM in physiological condition and HCM

### 2.1 Collagens

Collagens are the most abundant and well-studied ECM proteins in the heart (Eghbali and Weber, 1990; Ricard-Blum, 2011). They are deposited in the ECM and play structural roles contributing to mechanical properties, organization, and shape of tissues (Eghbali and Weber, 1990; Ricard-Blum, 2011; Villarreal and Dillmann, 1992). They interact with cells via several receptor families and regulate their proliferation, migration, and differentiation (Ricard-Blum, 2011).

#### 2.1.1 Fibril-forming collagens

Collagen I, the most abundant type of collagen in the heart and provides tensile strength to the myocardium as a fundamental part of the fibrous tissue in the cardiac interstitium (Ricard-Blum, 2011; Lombardi et al., 2003). Collagen III: often found in association with collagen type I and contributes to the elasticity of the myocardium (Ricard-Blum, 2011; Nikolov and Popovski, 2022). Collagen V: plays a role in regulating the fibril diameter and assembly of collagen fibers (Yokota et al., 2020). Though other collagen components have not been primarily found in the myocardium, it's worth noting that collagen composition in various tissues can be complex, and in certain pathological conditions or specific developmental stages (Ricard-Blum, 2011; Lombardi et al., 2003), the presence of other collagens in the myocardium might be investigated.

#### 2.1.2 Basement membrane (BM)

Collagen IV is a major component of the BM and represents a type of network forming collagens (Bruggink et al., 2007). It provides support and separates different tissue layers within the heart (Hynes and Naba, 2012; Ricard-Blum, 2011). Under normal physiological conditions, collagen serves to offer tensile strength, supporting the structural framework of myocytes, myofiber bundles, and sheets (Bruggink et al., 2007). This support is crucial for maintaining the normal functioning of the heart, and various cardiac disorders are linked to disturbances in the collagen matrix, such as its accumulation, depletion, or restructuring (Bruggink et al., 2007).

Several recent studies have highlighted the alteration of myocardial collagen upon myocardial hypertrophy, particularly with the hallmark of an increased interstitial fibrosis, which is considered an early manifestation of the diseases and mainly composed of fibrous collagen, influencing the stiffness of the myocardium, hindering the proper function of the heart (Ho et al., 2010; Díez et al., 2020). Alterations in myocardial collagen in HCM have been reported in several studies, with particular emphasis on collagen I and III, not only in the myocardium but further in the circulation as byproducts of collagen turnover, such as PIIINP (collagen III synthesis), PICP, PINP (collagen I synthesis) and ICTP (collagen I degradation), which both showed relevance to HCM phenotype, however data is not yet conclusive (Nikolov and Popovski, 2022; Ellims et al., 2014). Other collagens were also examined in HCM patients; collagen IV has showed alterations in expression pattern in HCM with a discontinuous and destroyed basal lamina (Bruggink et al., 2007; Ibrahim et al., 2020a), and exhibited an increased serum level, in correlation to fractional shortening and end-diastolic volume (Bruggink et al., 2007). Collagen V was recently reported to be downregulated in cardiac fibroblasts (CFs) in HCM (Ibrahim et al., 2022a), suggested to regulate the size of heart scars in an integrin-dependent manner (Yokota et al., 2020). However, collagen V protein has been reported to increase in HCM tissue specimens, alongside collagen I and VI (Previs et al., 2022).

Further, imaging techniques including cardiac magnetic resonance imaging (CMR), Cardiac CT scan, and Echocardiography have been able to quantify collagen content and assess myocardial fibrosis in HCM patients (Ho et al., 2010; Piers et al., 2013; Marwick and Narula, 2010), of which studies aimed to link CMR data to histological data for a more accurate representation of interstitial fibrosis in HCM patients (Diao et al., 2016; Espeland et al., 2018; Haaf et al., 2016). Increased collagen turnover and myocardial fibrosis and Left Ventricular Stiffness are associated with diastolic dysfunction in HCM (Ho et al., 2010). Enhanced collagen turnover contributes to increased left ventricular stiffness, impacting the functional capacity of the heart in HCM (Lombardi et al., 2003). The extent of collagen turnover and fibrosis is associated with the severity of symptoms in HCM, including heart failure symptoms (Olivotto et al., 2008). Of note, myocardial fibrosis resulting from altered collagen turnover is linked to the occurrence of arrhythmias in HCM patients (O’Hanlon et al., 2010). Indeed, collagen turnover in the myocardium involves a dynamic balance between collagen synthesis and degradation processes (Lombardi et al., 2003). Several factors and signaling pathways contribute to the control of collagen turnover, directly or indirectly, in the myocardium, such as Transforming Growth Factor-β (TGF-β) Signaling, Matrix Metalloproteinases (MMPs) and Tissue Inhibitors of Metalloproteinases (TIMPs), Angiotensin II Signaling, inflammation, other ECM proteins and other understudied factors such as miRNAs. Understanding the intricate regulation of collagen turnover in the myocardium is crucial for developing targeted therapies to modulate fibrosis and prevent adverse cardiac remodeling (Maron, 2002).

### 2.2 Proteoglycans

Proteoglycans are complex molecules composed of a core protein and long chains of glycosaminoglycans (GAGs) (Hynes and Naba, 2012; Rienks et al., 2014). They play essential roles in the ECM, contributing to tissue structure, cell signaling, and various physiological processes. In the myocardium, proteoglycans are crucial for maintaining the structural integrity of the cardiac ECM and influencing cell behavior (Hynes and Naba, 2012; Wang et al., 2019). The expression of proteoglycans in the myocardium is tightly regulated and can be influenced by various physiological and pathological conditions (Wang et al., 2019; Barallobre-Barreiro et al., 2021). In cardiac diseases such as myocardial infarction or heart failure, alterations in proteoglycan expression and remodeling of the ECM occur, impacting cardiac function. While not specific to HCM, proteoglycans expression has been reported to change in the aging heart, and some of the principles may apply to cardiac diseases (Christensen et al., 2019; Silva et al., 2020).

Hyalectans: Versican: a large chondroitin sulfate proteoglycan (CSPG) involved in tissue morphogenesis and inflammation (Sasi et al., 2023). It has been identified in the heart, where it influences cell adhesion and migration and has been known to be the major CSPG in the heart (Barallobre-Barreiro et al., 2021). It has recently been reported that versican is expressed after induction of pressure overload in mice, preceding collagen accumulation, particularly in collagen expressing CFs in transforming growth factor beta-dependent pathway (Sasi et al., 2023). Further, versican appeared to increase in the heart of HCM patients in conjunction with collagen increase, which suggests an involvement in the cardiac fibrosis (Previs et al., 2022; Sasi et al., 2023). Aggrecan: a large proteoglycan that forms giant hydrated aggregates with hyaluronan in the ECM is present in the heart and present in cardiac jelly, developing heart valves, and blood vessels during cardiovascular development, and contributes to the resilience and mechanical loading of the tissue (Krawetz et al., 2022). Mice with mutant aggrecan have been reported to have HCM (Koch et al., 2020). Further, Wistar rats undergone aortic banding, exhibited an increase in aggrecan mRNA, along with versican and other proteases (Vistnes et al., 2014). Aggrecan has also been reported for its overexpression in the aneurysmal aortic walls, increasing interlamellar swelling pressure, and disorganizing the aortic wall’s microstructure (Barallobre-Barreiro et al., 2020), which is has recently been reported to be associated with HCM (Ibrahim et al., 2022b). BM proteoglycans: Perlecan: A heparan sulfate proteoglycan, found in the BM and participates in cell-matrix interactions and helps regulate growth factor activities (Sasse et al., 2008; Johnson et al., 2024). Altered expression of Perlecan has been observed in cardiac diseases, including HCM, where it may contribute to abnormal cell-matrix interactions and affect growth factor signaling (Johnson et al., 2024). Perlecan null mice, had a severe effect on laminin and collagen IV, components of BM, compared to controls, and further exhibited a more severe dysfunction upon myocardial infarction, due to impaired BM composition and cardiomyocytes crosstalk with surrounding stroma and ECM (Sasse et al., 2008). Of interest, human pluripotent stem cell-derived cardiomyocytes (hPSC-CMs) cultured on a Perlecan substrate have exhibited hypertrophy and show heightened nucleation, characteristic of hypertrophic growth (Johnson et al., 2024). Interestingly, Perlecan appears to exert an opposing influence compared to Agrin, fostering cellular maturation instead of hyperplasia and proliferation (Johnson et al., 2024).

Cell membrane proteoglycans: Syndecan: Syndecans are a family of transmembrane heparan sulfate proteoglycans that play important roles in the myocardium, contributing to various cellular processes and tissue functions, such as Cell-ECM interaction, signal transduction, regulating CMs function, angiogenesis and tissue repair and remodeling (Lunde et al., 2016). Syndecans, particularly syndecan-4, are also involved in cardiac development, where they regulate signaling pathways involved in heart development, including Wnt, FGF, and BMP signaling (Mathiesen et al., 2019). A recent investigation into syndecan-4 underscored its significance in triggering the Ca^2+^-dependent calcineurin-NFAT signaling pathway, leading to hypertrophic remodeling and dysfunction in CMs under pressure overload conditions (Mathiesen et al., 2019; Lunde et al., 2022). Additionally, syndecan-4 has been reported to mediate muscle LIM protein nuclear translocation in CMs, a mechanism associated to HCM and dilated cardiomyopathy (DCM) (Mathiesen et al., 2019). Further, mice lacking syndecan-4 exhibited less collagen cross linking and fibrosis (Herum et al., 2015; Finsen et al., 2011), and further exhibited diminished capillary density, reduced cardiomyocyte size, and deteriorated left ventricular cardiac function following transverse aortic constriction (Li et al., 2017). Further, Syndecan‐4 was found to bind to osteopontin in LV and CFs protecting over deposition of collagen fibers (Herum et al., 2020). Interestingly, serum syndecan-4 has been shown potential as a new diagnostic and prognostic biomarker for LV remodeling in failing hearts (Takahashi et al., 2011).

Small Leucine Rich proteoglycans: Decorin is a small leucine-rich proteoglycan is expressed in the heart and is involved in collagen fibrillogenesis and interacts with various growth factors (Merline et al., 2009). Decorin has been suggested to induce cardiac hypertrophy by regulating the CaMKII/MEF-2 signaling pathway (Yang et al., 2021). However, other reports showed that Decorin overexpression can inhibit hypertension-induced cardiac fibrosis and hypertrophy and improved cardiac function (Yan et al., 2009), and can further inhibit TGF-β pathway and its pro-fibrotic effects on the failing human heart (Yan et al., 2009; Jahanyar et al., 2007), which makes it a potential candidate in HCM pathogenesis. Lumican (LUM) is a keratan sulfate small leucine-rich proteoglycan (SLRP) localized to the ECM, and known to regulate collagen fibrillogenesis in connective tissues, e.g., cornea, tendon and skin (Nikitovic et al., 2008). LUM is abundant in fibrotic tissues including the thickened intima of human atherosclerotic coronary arteries and is present in the developing myocardium (Mohammadzadeh et al., 2019; Hultgårdh-Nilsson et al., 2015). It has previously shown that LUM levels are increased in hearts of mice and patients with heart failure, via mediation cardiac remodeling, fibrosis, and inflammation (Mohammadzadeh et al., 2019; Mohammadzadeh et al., 2020), and accumulates with collagen fibers during HCM (Rixon et al., 2023). Proteomic analysis of myocardial specimens of HCM patients has shown that LUM is upregulated, correlating with the left atrial area myocardial fibrosis and the presence of a pathogenic sarcomere mutation (Coats et al., 2018), however this expression is yet debatable whether it could have a cardio-protective function (Guo et al., 2023).

### 2.3 Glycoproteins

Fibronectin (Fn) is a glycoprotein found in the ECM of tissues and plays a crucial role in various cellular processes, such as cell adhesion, migration, and signaling (Früh et al., 2015). In the myocardium, Fn contributes to the structural integrity of the ECM and participates in the regulation of cardiac development, remodeling, and repair (Talman and Ruskoaho, 2016). During embryonic development, Fn is expressed in the developing heart, where it contributes to the formation of the cardiac ECM (Jallerat and Feinberg, 2020). In adult myocardium, Fn is present in the ECM of the normal adult myocardium, where it forms a network that interacts with other ECM components, including collagen and proteoglycans (Chute et al., 2019). It further serves as a substrate for cell adhesion, allowing cells to attach and interact with the ECM via integrins and BM proteins (Farhadian et al., 1996). In response to cardiac injury, Fn expression can be upregulated in the myocardium in association with collagen deposition and TGF-beta 1 signaling (Villarreal and Dillmann, 1992; Wi et al., 1991). During myocardial fibrosis, there may be an excessive deposition of Fn as part of the fibrotic response (Piek et al., 2016). It has been reported that Fn contributes to pathological cardiomyocyte hypertrophy *in vitro* and *in vivo* via Nuclear Factor of Activated T cells activation (Konstandin et al., 2013) or via integrin beta 1-dependent activation (Chen et al., 2005). Further, Fn signaling is thought to stimulate BNP secretion, a gold standard indicator of HCM and cardiac fibrosis (Hasegawa et al., 1993), accompanied by hypertrophic responses *in vitro* (Ogawa et al., 2002). Of interest, circulating levels of fibronectin have been reported to be reduced in patients with HCM (Fucikova et al., 2016; Moretti et al., 2007), which raises the question of whether Fn in the circulation correlates to the myocardial expression in HCM.

Laminin (LN) is an essential component of the BM providing the integrity and function of CMs and blood vessels within the heart (Oliviéro et al.). It also contributes to the structural framework of the myocardium, specifically in anchoring CMs to the ECM, facilitating cell-to-cell communication, and contributing to the overall mechanical stability for cardiac tissues homeostasis (Oliviéro et al., 2000; Schwach and Passier, 2019). In hypertrophied CMs, LN was thought to contribute to alterations in sarcolemmal properties (Oliviéro et al., 2000), and its deficiency can lead to malformation in the myocardial microvasculature and subsequent ischemia, represented in elevated levels of hypoxia-inducible factor 1α (Hif1α) and vascular endothelial growth factor A (VEGFA) transcripts (Wang et al., 2006). Of note, mutation in the laminin alpha4 chain results in an abnormal myocardial ECM and subsequent muscle hypertrophy (Wang et al., 2006).

Fibulins (FBLNs) are a family of glycoproteins involved in ECM assembly and stabilization in different biological systems (Timpl et al., 2003). The widespread distribution of FBLNs correlates with their broad binding repertoire for fibronectin, collagens, BM proteins, elastin and proteoglycans (Timpl et al., 2003; Argraves et al., 2003). FBLN 1 and 2 are highly expressed in migratory cardiac mesenchymal during cardiac valvular septal formation (Argraves et al., 2003; Cooley et al., 2008), which dragged attention to their role in cardiac development and further in pathological conditions such as HCM. While research on FBLNs in HCM is not extensive, recent reports indicated that FBLN2 plays an essential role in Ang II-induced TGF-β signaling and subsequent myocardial fibrosis (Khan et al., 2016). FBLN4 was reported to be crucial for elastic fiber formation (Halabi et al., 2017), and mutations in the *FBLN4* gene have been associated with aortic aneurysms and dissections (Loeys et al., 2005). FBLN5 is also involved in elastic fiber assembly and is expressed in various tissues, including the heart (Chapman et al., 2010; Wang et al., 2005). Knowledge on FBLN5 in the context of HCM specifically is limited, nonetheless, its role in ECM maintenance suggests potential implications for cardiac remodeling (McLaughlin et al., 2007). Of interest, we recently reported that CF-associated transcriptomics signature comprised upregulation of FBLN1 and FBLN5 genes, which was further confirmed in the tissues of HCM patients (Ibrahim et al., 2020a). We further reported that FBLN2, which has common binding partners with FBLN1 and FBLN5, is upregulated in the CMs and the circulation of HCM patients, however, protein expression in CFs did not significantly change; an observation that was further confirmed by our generated transcriptome signature of HCM-CFs (Ibrahim et al., 2020a). Further, it has been suggested that FBLN5 modulate TGF-β signaling, a pathway implicated in tissue fibrosis and remodeling (Nakasaki et al., 2015), hallmarks of HCM. It has also been reported that FBLNs 1, 2 and 5 are reduced in the aorta of HCM patients, in association with an increase in aortic stiffness, which introduce FBLNs as targets for cardiac and extra-cardiac tissues (Ibrahim et al., 2022b).

Periostin is a member of the glycoprotein family (Stansfield et al., 2009). Studies have indicated the significant involvement of periostin in fostering collagen fibrogenesis and promoting a fibroblastic lineage during the maturation of atrioventricular valves in cardiac development (Norris et al., 2008; Norris et al., 2009). While its expression remains low in adult hearts, periostin is crucial for maintaining the biomechanical characteristics of mature myocardium (Kühn et al., 2007). Periostin has been shown to correlate and contribute to cardiac remodeling and fibrosis in overloaded hearts and heart failure (Zhao et al., 2014; Ioakeimidis et al., 2023). Interestingly, periostin has been shown to mediate the AngII via ERK1/2 and TGF-β1/Smad signaling (Li et al., 2011). Nevertheless, some reports showed that periostin might be involved in the transdifferentiation of CMs leading to cardiac repair (Kühn et al., 2007). The distribution and expression patterns of periostin, which correlated with the degree of myocardial fibrosis, could serve as a potential biomarker for cardiac remodeling in patients with HCM heart failure (Zhao et al., 2014).

Tenascin-C (TNC) is a glycoprotein categorized as a matricellular protein and exhibits transient expression patterns at various crucial stages of embryonic heart development (Imanaka-Yoshida et al., 2020; Tucker and Chiquet-Ehrismann, 2009). In the normal adult heart, its presence is minimal, yet under pathological conditions linked to inflammation, such as myocardial infarction, hypertensive cardiac fibrosis, myocarditis, dilated cardiomyopathy, and Kawasaki disease, TNC is re-expressed in a spatially and temporally confined manner (Imanaka-Yoshida et al., 2020; Tucker and Chiquet-Ehrismann, 2009). It has recently been reported that upon myocardial infarction, interstitial cells located in the border zone begin producing TNC serving to weaken the adhesion between surviving CMs and ECM, potentially facilitating the reorganization of the tissue (Imanaka-Yoshida et al., 2001). TNC has also demonstrated the ability to induce inflammatory reactions by hastening the migration of macrophages and the production of proinflammatory and profibrotic cytokines through the integrin αVβ3/FAK-Src/NF-κB pathway, leading to an increased fibrosis (Shimojo et al., 2015). TNC has been reported to prompt cardiac myocytes to enhance the activation of genes linked to hypertrophy and MMPs (Podesser et al., 2018). Conversely, removing TNC could lessen the inflammatory and fibrotic changes, as well as hypertrophy, and diminish contractile dysfunction in hearts undergoing TAC (Podesser et al., 2018). Of interest, serum TNC has shown a prognostic power in HCM patients (Kitaoka et al., 2012).

### 2.4 Proteases and their inhibitors: MMPs, ADAMs and TIMPs

MMPs are enzymes responsible for collagen degradation, while TIMPs inhibit MMP activity (Nagase et al., 2006). The balance between MMPs and TIMPs influences collagen turnover (Spinale, 2007; Cambronero et al., 2009). Of the known MMPs in the myocardium are: MMP-1 and MMP-8, which are involved in the degradation of type I and type III collagens (which are classical major components of the myocardial fibrillar collagen and interstitial fibrosis) (Takahashi et al., 1999). MMP-2 (Gelatinase A), which is involved in the degradation of type IV collagen (Spinale, 2007), MMP-9 (Gelatinase B), which is involved in the degradation of type IV collagen and is associated with tissue remodeling and inflammatory processes (Yabluchanskiy et al., 2013), MMP-3 (Stromelysin-1), which participates in the breakdown of fibronectin and laminin (Rodríguez et al., 2010), MMP-13 (Collagenase-3), which targets type II collagen (Takahashi et al., 1999; Uesugi and Sakata, 2005), and MMP-14 (MT1-MMP), which plays a crucial role in ECM remodeling and activates other MMPs, contributing to tissue homeostasis (Nagase et al., 2006). In pathological cardiac remodeling, a group of MMPs such as MMP-2 and MMP-9, are upregulated, leading to increased ECM degradation and subsequent fibrosis (Roldán et al., 2008; Takawale et al., 2017). and their levels in plasma were associated to NT-proBNP levels and further related to clinical parameters such as LV ejection fraction, LV end-diastolic dimension, exercise capacity and the maximum LV wall thickness (Roldán et al., 2008; Kitaoka et al., 2011; Bi et al., 2021). Although MMPs have been associated with myocardial fibrosis, MMP1 has been reported to attenuate the development of cardiac fibrosis in mouse models (Foronjy et al., 2008), however other studies reported the increase of circulating MMP1 levels in HCM patients (Fernlund et al., 2017). Therefore, understanding the pathophysiology mechanisms of MMPs, and cell-specific MMPs and (Ibrahim et al., 2022a; Toba et al., 2017), is crucial for identifying personalized targeting approaches.

TIMPs, on the other hand, act as inhibitors and regulators of MMPs (Nagase et al., 2006). TIMP-1, is a broad-spectrum inhibitor of MMPs and primarily inhibits MMP-1, MMP-2, MMP-3, and MMP-9 (Brew and Nagase, 2010), TIMP-2, inhibits a range of MMPs, including MMP-1, MMP-2, MMP-3, and MMP-9 and is also involved in regulating cell growth and apoptosis (Stetler-Stevens and on, 2008), TIMP-3, has a broader inhibitory profile, affecting MMP-1, MMP-2, MMP-3, MMP-9, and ADAMs (a disintegrin and metalloproteinases), and plays a crucial role in maintaining tissue integrity and inhibiting angiogenesis (Brew et al., 2000), and TIMP-4 inhibits MMP-2 and MMP-9 and plays a role in modulating tissue responses to injury and inflammation (Cabral-Pacheco et al., 2020). Circulating TIMP1 and TIMP2 were reported to be increased in HCM, in association with LV end‐systolic dimension, Left atrium dimension, and LV ejection fraction (Kitaoka et al., 2010). Of interest, a recent study has shown that TIMP1 deficiency have significantly reduced myocardial fibrosis via meditating an association between CD63 (cell surface receptor for TIMP1) and integrin β1 on CFs, leading to *de novo* collagen synthesis, reducing myocardial fibrosis, independent from MMPs (Takawale et al., 2017).

Besides, ADAMs are membrane-anchored proteins that mediate ectodomain shedding of substrate proteins, and play diverse roles in the normal myocardium, including cell adhesion, proteolysis, and signaling, however, their exact role requires further investigation (Weber and Saftig, 2012). ADAM12 for instance, mitigates the excess hypertrophic response by attenuating integrin-mediated downstream signaling (Nakamura et al., 2020).

Further, activation of protease-activated receptors (PARs) by proteases, such as thrombin, has been implicated in cardiac hypertrophy (Antoniak et al., 2011). PARs may contribute to signaling pathways that influence hypertrophic responses (Antoniak et al., 2011).

### 2.5 Cytokines and chemokines

Cytokines are small signaling proteins that play crucial roles in the regulation of immune responses, inflammation, tissue repair, remodeling, and adaptation to various physiological stimuli (Hanna and Frangogiannis, 2020). While the heart is traditionally viewed as an organ with limited immune activity, it does produce and respond to certain cytokines under healthy conditions (Hanna and Frangogiannis, 2020; Mann, 2015). In physiological conditions, the myocardium maintains a balanced and regulated environment, and the expression of cytokines is generally at low levels (Fang et al., 2017). In HCM however, pro-inflammatory cytokines are elevated, contributing to fibrosis and ECM alterations, which has been suggested to compose a chronic “low grade” inflammatory microenvironment (Hanna and Frangogiannis, 2020; Lillo et al., 2023). Interleukin-10 (IL-10) and IL-1β are pro-inflammatory cytokines that are involved in immune responses and inflammation (Xu et al., 2021). In physiological conditions, their expression in the heart is generally low, only sufficient to help regulate immune responses and reduce inflammation (Xu et al., 2021). In HCM, IL-10 may play a protective role by modulating inflammatory responses and attenuating myocardial remodeling (Sziksz et al., 2015). IL-1β is involved in inflammatory responses and may contribute to the progression of cardiac hypertrophy in HCM (Schwinger et al., 1994).

**FIGURE 3:**
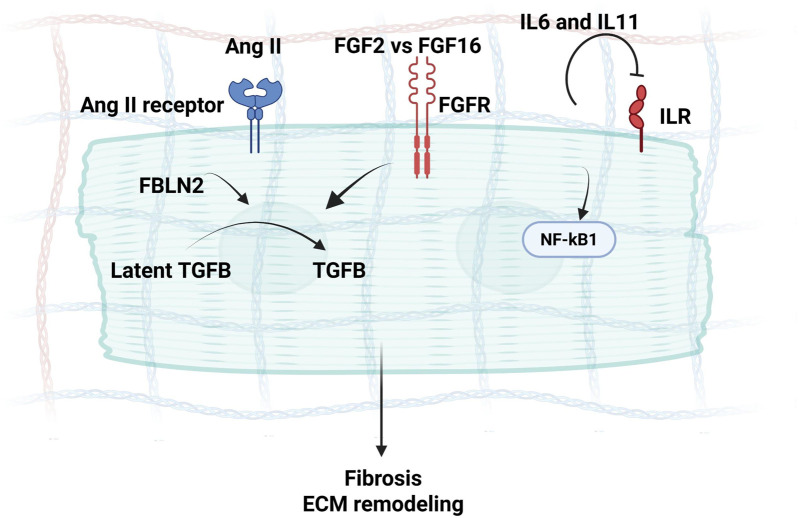
A schematic diagram demonstrating secretome mediators of Ang II signaling in CMs during interstitial fibrosis and Hypertrophy, where Fibulin-2 can mediate Angiotensin signaling via the activation of TGF-β, along with the concurrent FGF and IL6 signaling, inducing myocardial fibrosis.

TGF-β is a multifunctional cytokine involved in maintaining tissue integrity and preventing excessive inflammation in the myocardium (Hanna and Frangogiannis, 2019). In HCM, TGF-β signaling has been long known for its upregulation in experimental models of myocardial infarction and cardiac hypertrophy (Frangogiannis, 2020; Teekakirikul et al., 2010). Endogenous TGF-β plays a crucial role in the development of cardiac fibrotic and hypertrophic remodeling, as well as in regulating ECM metabolism in the pressure-overloaded heart (Frangogiannis, 2020). TGF-β deactivates inflammatory macrophages while facilitating myofibroblast transdifferentiation and ECM synthesis through Smad3-dependent pathways (Saadat et al., 2020). Consequently, TGF-β may function as the pivotal “master switch” orchestrating the transition from the inflammatory phase to scar formation in the infarcted heart (Rienks et al., 2014), and activate Angiotensin II signaling (via FBLN2 mediation) (Zhang et al., 2014) (Figure 3), all of which are hallmarks of HCM. Efforts aimed at translating these concepts into therapeutic approaches to mitigate cardiac hypertrophy and fibrosis face challenges due to the intricate, multifaceted nature of TGF-β signaling (Hanna and Frangogiannis, 2019). Concerns arise regarding the potential harmful effects of inhibiting TGF-β and the possibility of limited benefits for patients already receiving optimal treatment with ACE inhibitors and β-adrenergic blockers (Lim et al., 2001; D et al., 2011).

IL-6 has both pro-inflammatory and anti-inflammatory properties, and may contribute to normal physiological processes, such as the response to exercise and stress (Scheller et al., 2011). IL-6 has been associated with inflammation and cardiac hypertrophy in HCM and increased IL-6 levels may contribute to disease progression (Teekakirikul et al., 2010). IL-6 has been found to be essential in increasing collagen content regulated by isolated CFs and played a role in mediating a phenotypic conversion to myofibroblasts, via Angiotensin II induction (Meléndez et al., 2010). It has also been reported for its mediation to the Angiotensin II signaling during cardiac hypertrophy (Gro et al., 2019; Högye et al., 2004). Higher IL-6 levels in the both the myocardium and the circulation have been associated with larger infarct size and decreased cardiac function in HCM (Gro et al., 2019; Högye et al., 2004).

Tumor Necrosis Factor-Alpha (TNF-α) is a pro-inflammatory cytokine that is typically associated with immune responses and plays a role in adaptation to exercise (Jang et al., 2021). (Xu et al., 2021)TNF-α has been reported to contribute to myocardial dysfunction (Tian et al., 2015), with an association of higher expression along with IL6, with HCM (Sano et al., 2000). CMs-specific expression of TNF-α has shown to lead to LV hypertrophy (Schumacher and Naga Prasad, 2018), however, studies have shown contradiction of TNF-α effect based on its source (Miao et al., 2020; Yokoyama et al., 1997; Feldman et al., 2000). Of interest, inhibition of TNF-α reduces adverse myocardial remodeling in a rat model of volume overload (Jobe et al., 2009). Another recently studied interleukin in HCM is IL11, which is a member of the IL6 family, and its receptors are mainly expressed in CFs (Alter et al., 2023). In IL11-stimulated CFs, collagen, ECM remodeling components such as periostin and MMP2 are strongly upregulated at the protein level (Sweeney et al., 2020). Blocking of IL11 signaling with Lutein has recently been suggested to attenuate angiotensin II- induced cardiac remodeling and fibrosis (Chen et al., 2021) (Figure 3). As a biomarker, elevated plasma IL-11 levels have been associated with a notable rise in cardiac events and indicate a poor prognosis in HCM and heart failure patients (Ye et al., 2019). On the other hand, chemokines, such as monocyte chemoattractant protein-1 (MCP-1), play a role in recruiting immune cells to the heart during inflammation, which can subsequently impact ECM homeostasis (Sun et al., 2021). MCP-1/CCL2 is associated with the recruitment of monocytes and macrophages to the site of inflammation (Shen et al., 2014). Myocardial and circulating MCP1 levels have been reported to increase in HCM patients particularly in patients with systolic dysfunction (Iwasaki et al., 2009). MCP1-driven pro-inflammatory signaling may accentuate cardiomyocyte death and can mediate fibrosis upon recruiting monocytes and macrophages that secrete mediators, such as TGF-β (Hanna and Frangogiannis, 2020), a key driver in myocardial fibrosis in HCM (Ibrahim et al., 2020a). Of interest, *in vitro* experiments have revealed that a combination of IL-6 with MCP1 sustained STAT3 activation in CMs, promoting the differentiation of CFs into myofibroblasts under hypoxic conditions (Morimoto et al., 2006). In agreement, we have recently reported that CCL2 is overexpressed in HCM CFs and myocardium in association with IL6 and other pro-inflammatory drivers, such as CCL11 and CCL4 (Ibrahim et al., 2022a).

Osteopontin (OPN) is a matricellular protein that mediates diverse biological functions and functions as a proinflammatory cytokine promoting cell-mediated immune responses (Shirakawa and Sano, 2021). OPN has been implicated in the progression of fibrosis induced by Ang II (Mohamed et al., 2019; Matsui et al., 2004), a key driver of interstitial fibrosis in HCM (Zhang et al., 2014). It has exhibited interactions with diverse ECM proteins such as fibronectin and collagen, indicating its potential involvement in organizing and stabilizing the matrix structure (Matsui et al., 2004). Lack of OPN could potentially decrease the rise in blood pressure induced by Ang II and improve the progression of cardiac fibrosis (Matsui et al., 2004). It has therefore been suggested as a therapeutic target for HCM and heart failure for its role in cardiac fibrosis (Mohamed et al., 2019).

Fibroblast growth factors 2 and 16 (FGF2 and FGF16): FGFs are proteins that serve a variety of functions in the tissue development, repair, and metabolism (Itoh and Ornitz, 2008). FGF16 stands out among paracrine FGFs as it is predominantly expressed in cardiac tissue (Itoh and Ohta, 2013; Hotta et al., 2008). While FGF16 expression is relatively low in the embryonic heart, it becomes more abundant during adulthood compared to embryonic stages, which suggest potential roles for FGF16 in cardiac function (Tacer et al., 2010). A recent study on a mouse model, has shown that FGF16 prevents angiotensin II-induced cardiac hypertrophy and fibrosis by antagonizing FGF2 (Matsumoto et al., 2013). Further, deleting FGF2 attenuates muscle hypertrophy in adult mice (Schultz et al., 1999). We have recently reported CF-specific upregulation of FGF16 and downregulation of FGF2 in HCM patients. Nonetheless, the interplay between FGF16 and FGF2 in the cardiac tissue microenvironment is yet debatable and arise from their competition on FGFR to activate MAPK signaling and induce tissue remodeling (Itoh and Ohta, 2013).

### 2.6 Integrins

Integrins: Integrins play several crucial roles in the myocardium, serving as key mediators of cell-cell and cell-ECM interactions (Ross and Borg, 2001). They can be expressed on either CFs or CMs, mainly for mediating the interaction between them and the ECM, particularly collagen (such as integrins α1β1, α2β1, α11β1) (Ross and Borg, 2001; Mezu-Ndubuisi and Maheshwari, 2020; Harston and Kuppuswamy, 2011). Integrins are also involved in Mechanical Signaling bidirectionally between the ECM and the intracellular cytoskeleton, which is essential for regulating cellular processes such as cell contraction, proliferation, and gene expression in response to changes in mechanical forces (Ross and Borg, 2001; Israeli-Rosenberg et al., 2014). Signal Transduction via activating intracellular signaling pathways in response to ECM ligands, for cell survival, proliferation, differentiation, and gene expression (Harston and Kuppuswamy, 2011; Ross, 2002). Angiogenesis via mediating the adhesion and migration of endothelial cells (ECs), which are essential for the formation of new blood vessels during myocardial development, tissue repair, and ischemic injury (Mezu-Ndubuisi and Maheshwari, 2020). Electrical Coupling between CMs and the ECM, contributing to the transmission of electrical signals between cells and modulating cardiac conduction properties (Valencik et al., 2006; Dabiri et al., 2012).

Several *In vitro* and *in vivo* models have studied the association of integrins with cardiac hypertrophy (Harston and Kuppuswamy, 2011). In the pathological myocardium, expression of the integrins isoforms is altered leading to alterations in CFs, the ECM and CMs, and in response to the mechanical stretch resulting from hypertrophy (Brancaccio et al., 2006). Integrin pathological signaling may result in the activation of myofibroblasts or the development of CM hypertrophy (Maitra et al., 200). Deletion of β1 integrin in mice has been reported to reduce myocardial proliferation and impaired ventricular compaction. (Maitra et al., 200).Interestingly, it has been recently reported that Integrin beta-like 1 is an important functional mediator between fibroblast–cardiomyocyte crosstalk and could be an effective target for cardiac remodeling in myocardial hypertrophy and HCM (Chen et al., 2023), particularly with its reported interaction with multiple ECM proteins during myocardial remodeling (Laser et al., 2000).

## 3 Interactions between matrisome components in the myocardium

As previously highlighted, the matrisome comprises a network of core ECM proteins (e.g., collagens, proteoglycans, glycoproteins) and matrisome-associated proteins (e.g., ECM regulators, affiliated proteins, and secreted factors), which interact to determine the structural and signaling microenvironment of the myocardium. In HCM, dysregulation of these interactions contributes to pathological fibrosis, impaired mechano-transduction, and chronic inflammation (Dabiri et al., 2012; Martino et al., 2018). For example, fibronectin interacts with collagen I and III via specific domains to promote fibrillogenesis and scaffold assembly (Singh et al., 2010), while decorin and lumican regulate collagen fiber diameter and cross-linking, modulating tissue stiffness (Chen et al., 2020). Perlecan binds laminin and collagen IV in the basement membrane, supporting endothelial cell adhesion and barrier function (Yousif et al., 2013). Proteoglycans like versican form large aggregates with hyaluronan, facilitating hydration and influencing leukocyte infiltration during inflammation. Furthermore, fibulins serve as bridging molecules, linking elastin, collagen, and glycoproteins like fibronectin, and modulating growth factor availability such as TGF-β sequestration (Sasi et al., 2023). Further, Fibulins were repeatedly reported to interact with each other’s and with BM proteins such as laminin and Col IV (Ibrahim et al., 2018; Olijnyk et al., 2014; WalyEldeen et al., 2024).

These protein–protein interactions are dynamic and context-dependent, influenced by post-translational modifications, mechanical cues, and localized cellular and molecular activity in the myocardium. Their disruption or overactivation in HCM alters ECM organization, leading to increased myocardial stiffness, altered electrical conductivity, and myocyte-ECM uncoupling. Therefore, dissecting the physical and biochemical interplay between matrisome components may offer new insights into the progression of HCM and the identification of matrix-based therapeutic targets.

## 4 Sources of ECM components

**FIGURE 4:**
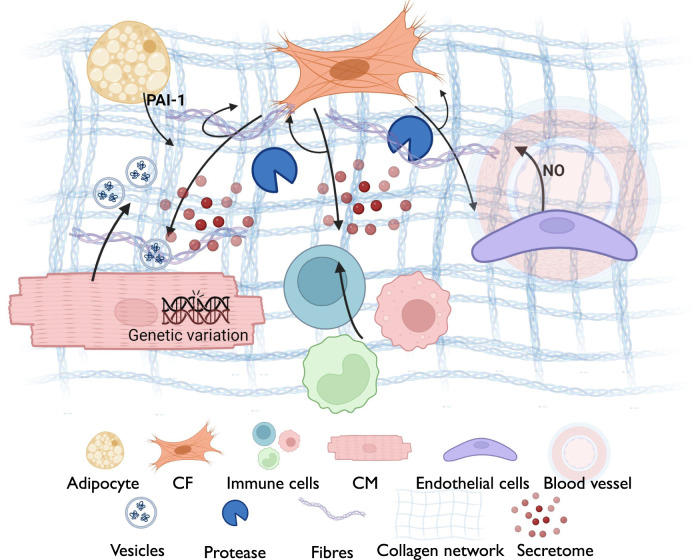
A schematic diagram summarizing the cross-talk between different myocardial components for ECM remodeling, including the reciprocal interaction between cardiomyocytes (*Fibronectin; thrombospondin-4; ECM-interacting proteins (integrins); MMPs; exosomes with ECM-modulatory factors*) and cardiac fibroblasts *(Collagen types I, III, V; fibronectin; periostin; proteoglycans (decorin, biglycan); TGF-β; MMPs and TIMPs*), immune cells *(TGF-β, IL-1β, IL-6, IL-11; matrix-degrading enzymes (MMPs); osteopontin*), adipocytes (*Adipokines (leptin, resistin); pro-fibrotic cytokines; influence on fibroblast ECM secretion*) and endothelial cells (*Basement membrane proteins (collagen IV, laminin, perlecan); regulators of angiogenesis (angiopoietin, VEGF*). This interaction is governed by several factors including the genetic variation, secreted components from various cell types and the ECM network surrounding the cellular niche.

The human heart consists of five primary cell types: CMs, CFs, ECs and immune cells such as macrophages, and adipocytes (Hall et al., 2021). The dynamic interplay between various cell types and their secreted products regulates the structural and functional properties of the myocardial ECM (Rienks et al., 2014). Understanding the sources and regulation of ECM components is crucial for deciphering the complex biology of the heart. Several sources contribute to the composition of the ECM in myocardial tissue (Figure 4).

### 4.1 Cardiac fibroblasts

CFs represent the major non-cardiomyocyte cell lineage that maintain the myocardial homeostasis and ECM turnover (Travers et al., 2017). They are the primary cell type responsible for producing and regulating the majority of the known ECM components (Fan et al., 2012; Travers et al., 2017). CFs are the main source of collagen, particularly collagen types I and III (Verdecchia et al., 2012; Kanisicak et al., 2016). They also contribute to the synthesis of Fn, MMPs and TIMPs (Fan et al., 2012; Travers et al., 2017). During myocardial injury and HCM, CFs become activated, via mechanical and/or molecular signaling, and may differentiate to myofibroblasts, with an expression of smooth muscle actin (SMA), increased secretion of inflammatory mediators, and increased deposition of ECM proteins (Marian and Braunwald, 2017; Fan et al., 2012), which represent stress responses that aggravate heart diseases (Fan et al., 2012). CFs-associated FGF2 and FGF16 contribute to cardiac hypertrophy via stimulating CMs in a paracrine fashion (Fujiu and Nagai, 2014). Further, interleukins such as IL6 and IL11 were reported to be secreted by CFs during cardiac injury and hypertrophy (Hall et al., 2021). CFs alternatively respond to inflammatory mediators and adipokines either via autocrine or paracrine loops, contributing to an inflammatory environment that influences ECM remodeling in HCM (Camelliti et al., 2005; Tallquist and Molkentin, 2017). Activated CFs exhibit dysregulated MMPs and TIMPs expression, influencing ECM turnover (Fan et al., 2012). They are involved in the activation of the TGF-β signaling pathway, which is associated with fibrosis and ECM remodeling in HCM (Fujiu and Nagai, 2014; Lasala et al., 2012).

The crosstalk between CFs and CMs, as well as with the surrounding stroma/ECM, is bidirectional and crucial for tissue homeostasis (Hall et al., 2021; Bursac, 2014). In pathological conditions, all these elements are influenced by altered signaling cascades, leading to a microenvironment that chronically affects CFs phenotype and relevant response (Hall et al., 2021). CM-associated signals such as TGF-β, angiotensin II, and microRNAs promoting CFs activation, myofibroblast transition, and increased collagen, fibronectin, and periostin synthesis. CFs have recently become targets for novel cardiac therapeutics due to their primary contribution to ECM remodeling, their direct interaction with CMs, and their ability to differentiate and regenerate (Hall et al., 2021). While CFs are the primary ECM-producing cells in the myocardium, the intricate crosstalk between CMs and other cell types in the heart contributes to the dynamic and finely tuned process of ECM remodeling (Camelliti et al., 2005; Bursac, 2014). Understanding these interactions is crucial for unraveling the complexities of cardiac physiology and pathology, particularly in conditions like myocardial infarction, hypertrophy, and heart failure.

### 4.2 Cardiomyocytes

While CMs are traditionally recognized for their contractile function in the heart, CMs also play a significant role in ECM remodeling (Rienks et al., 2014; Mouw et al., 2014). CMs can secrete fibronectin and MMPs, regulated, in part, by factors like mechanical stretch, cytokines, and neurohormones (Aoyagi and Matsui, 2011). CMs-Integrins mediate the interaction between CMs and the ECM which activates intracellular signaling pathways that can influence cell behavior, including gene expression related to ECM remodeling (Aoyagi and Matsui, 2011). Mechanical forces, such as those generated during contraction, can affect CMs behavior and gene expression, and can activate pathways that influence ECM synthesis and remodeling for maintaining tissue integrity and preventing adverse remodeling (Martino et al., 2018). CMs release extracellular vesicles, including exosomes, which can transport bioactive molecules including microRNAs, that influence neighboring cells, including CFs involved in ECM regulation (Aoyagi and Matsui, 2011). Adaptive Responses to Stress: Under conditions of stress, such as hypertrophy or ischemia, CMs can undergo adaptive changes that influence ECM remodeling. This may involve alterations in gene expression profiles that impact the synthesis and degradation of ECM components. Indeed, the genetic variations encompass HCM etiology, particularly those associated with sarcomere proteins (Marian and Braunwald, 2017; Allouba et al., 2023), accounts for the cellular and molecular alterations in CMs that can cause downstream alterations in the ECM (Dour et al., 2017).

### 4.3 Endothelial cells

Endothelial Cells are lining the blood vessels within the myocardium secrete various ECM components, including BM proteins such as laminin and collagen IV (Widyantoro et al., 2010). ECs can also secrete various ECM components, including fibronectin and laminins, which are important for maintaining the structural integrity of blood vessels and the surrounding tissue (Yousif et al., 2013). ECs are crucial for angiogenesis, which requires balanced ECM remodeling for vessel sprouting, branching, and stabilization (Gogiraju et al., 2019). ECs release various growth factors and cytokines that influence the behavior of neighboring cells, including CFs (Mai et al., 2013). These factors can modulate ECM turnover and remodeling (Davis and Senger, 2005; Bischoff et al., 2005). Of interest, ECs produce nitric oxide (NO), which has vasodilatory effects and plays a role in maintaining vascular tone (Bischoff et al., 2005). Dysregulation of NO production may influence ECM remodeling and contribute to vascular changes (Heiss et al., 2015). Of note, changes in the microvasculature, influenced by ECs, may impact nutrient and oxygen supply to the myocardium. These changes can have downstream effects on ECM homeostasis.

### 4.4 Immune cells

Various immune cells, including macrophages, play a role in tissue repair (Davies et al., 2013; Wang et al., 2020). While immune cells are primarily associated with the immune response and inflammation, their interactions with other cell types, including CFs and CMs, can influence ECM remodeling (Toba et al., 2017; Lavine et al., 2018). Immune cells can infiltrate the myocardium in response to various stimuli and contribute to ECM remodeling and has recently been suggested to halt myocardial fibrosis and promote angiogenesis (Revelo et al., 2021; O’Rourke et al., 2019). Immune cells release cytokines, which are signaling molecules that can influence the behavior of CFs and other cells involved in ECM maintenance (Frieler and Mortensen, 2015). An Altered cytokine expression, including pro-inflammatory cytokines, has been reported in HCM and may contribute to cardiac remodeling (Piek et al., 2016). As mentioned earlier, immune cells can produce MMPs and TIMPs, in response to pathological stimuli and in coordination with CFs and CMs (Lavine et al., 2018; Frieler and Mortensen, 2015). CFs may respond to signals from immune cells, influencing collagen synthesis and deposition (Hitscherich and Lee, 2021). This stimulation cascade varies based on tissue status. During acute myocardial infarction, pro-inflammatory cytokines are released to initiate inflammation and clear necrotic tissue. Once the necrotic tissue is removed, macrophages transition to an anti-inflammatory phenotype, stimulating CFs to deposit collagen and promote fibrotic tissue formation (Chen et al., 2024), a mechanism similarly observed in cancer models (Ibrahim et al., 2020b; Wang et al., 2020). This interplay is activated in various cardiac pathologies, including ischemia and pressure-overloaded myocardium, and is linked to the regulation of ECM proteins, such as MMPs (Chen et al., 2024; Waleczek et al., 2022), and can be orchestrated via myocardium-resident macrophages (Waleczek et al., 2022), or previously activated monocytes (Chen et al., 2024).

### 4.5 Adipocytes

Adipocytes are fat cells found in the myocardial tissue and can contribute to the ECM by secreting various adipokines and other signaling molecules (Chait and den Hartigh, 2020). The specific role of adipocytes in ECM maintenance in the myocardium, particularly in HCM, has not been extensively studied. Increased epicardial fat thickness has been associated with disease severity and adverse clinical outcomes (Hajsadeghi et al., 2014; Talman et al., 2014), such as atrial fibrillation and coronary heart conditions, which are complications of HCM (Macintyre and Lakdawala, 2016). Associations between epicardial adipose tissue volume and arrhythmias may have relevance to HCM patients with arrhythmic complications (Conte et al., 2022). Adipokines secreted by the adipose tissue, such as adiponectin and leptin, have the potential to influence the restructuring of the ECM in the myocardium, via regulating the expression of proteases (TIMPs and MMPs). plasminogen activator inhibitor type 1, primarily synthesized by adipose tissue, controls the function of plasmin, a serine protease crucial for regulating the ECM (Zibadi et al., 2011; Schram and Sweeney, 2008). Adipocytes can engage in paracrine signaling with neighboring cells, including CFs and CMs (Krishnan et al., 2021). The interplay between adipocytes and the cardiac microenvironment is an active area of research, and there are several considerations regarding their potential contributions.

## 5 Candidate myocardial ECM proteins with clinical relevance

A plethora of biomarkers have been defined in myocardial pathologies, in particular HF and HCM, and are associated with pathophysiological pathways in disease progression, such as markers of neurohormonal activation (ET-1) (Widyantoro et al., 2010), oxidative stress (Myeloperoxidase (MPO)), and myocyte injury and stress (cardiac troponins, Brain Natriuretic Peptide (BNP) and NT-ProBNP) (Ho et al., 2017; Captur et al., 2020). ECM remodeling biomarkers have recently been introduced to clinical research as an attempt to expand the phenotype screening of HCM, especially with the disease clinical heterogeneity. Inflammation and ECM-associated markers such as sST-2, TGF-B, TIMPs and MMPs, have been a focus of recent studies for that purpose (Matthia et al., 2022). Elevated levels of collagen turnover biomarkers—such as PIIINP, PICP, PINP, and ICTP correlate with fibrosis severity and phenotype differentiation, making them valuable non-invasive markers of ECM remodeling (Lombardi et al., 2003; Matthia et al., 2022). TGF-β is a central regulator of myocardial fibrosis and inflammation, linking molecular pathways to phenotypes and serving as an experimental therapeutic target despite the challenges posed by its pleiotropic nature (Hanna and Frangogiannis, 2020; Hanna and Frangogiannis, 2019; Matthia et al., 2022). The circulating levels of MMPs and TIMPs correlate with fibrosis severity and outcomes, offering diagnostic and therapeutic insights (Spinale, 2007; Matthia et al., 2022). Similarly, reduced fibronectin levels are associated with myocardial hypertrophy progression, and it has been explored for diagnostic correlation with BNP secretion and fibrosis, as well as a target for anti-fibrotic therapies (Konstandin et al., 2013; Matthia et al., 2022).

Other ECM proteins also contribute to fibrosis and hypertrophy in HCM; Lumican, identified through proteomic analyses, correlates with fibrosis severity and left atrial enlargement, and has been proposed as a marker of advanced fibrosis (Rixon et al., 2023) TNC is associated with inflammation-driven HCM and adverse outcomes, with serum levels offering prognostic value in heart failure (Kitaoka et al., 2012; Kitaoka et al., 2010; Matthia et al., 2022). Syndecan-4, involved in fibrosis progression and myocardial stiffness, is under investigation as both a biomarker and therapeutic target (Takahashi et al., 2011). OPN levels increase significantly with fibrosis and adverse remodeling, making it a biomarker and therapeutic target (Mohamed et al., 2019; Matthia et al., 2022). Having been intensively studied, interleukins, such as IL-6 and IL-11, stratify patients based on fibrosis and inflammation burden. IL-11 in particular predicts poor prognosis and has therapeutic potential (Gro et al., 2019; Högye et al., 2004; Sano et al., 2000; Ye et al., 2019; Cook, 2023). Lastly, fibulins—especially circulating Fibulin-2—are suggested to correlate with myocardial fibrosis in HCM, adding further value to the pool of ECM-related biomarkers in cardiac disease management (Ibrahim et al., 2020a).

## 6 *In Vitro* modeling and future directions

HCM is increasingly recognized not only as a disease of the sarcomere but also as a complex condition involving extensive remodeling of the myocardial ECM. The evidence presented in this review highlights that ECM components—including collagens, proteoglycans, glycoproteins, proteases, cytokines, and integrins—undergo substantial quantitative and qualitative changes that contribute to hallmark features of HCM such as interstitial fibrosis, diastolic dysfunction, and arrhythmogenesis. Recent advances in disease modeling have expanded our ability to explore these ECM changes with greater specificity and translational relevance. In particular, *in vitro* systems such as human induced pluripotent stem cell-derived cardiomyocytes (iPSC-CMs) and engineered heart tissues (EHTs) provide platforms to study the molecular and cellular interactions between ECM components and cardiac cells under genetic and biomechanical stress (Yildirim et al., 2025; Jebran et al., 2025). iPSC-CMs derived from HCM patients exhibit aberrant fibronectin deposition, BNP secretion, and ECM-associated signaling responses (e.g., TGF-β activation), allowing mechanistic dissection and therapeutic screening. Co-culture systems with CFs or immune cells further enable modeling of the cellular crosstalk driving ECM remodeling. These models, combined with high-content imaging and single-cell omics, offer insights into disease heterogeneity and therapeutic responsiveness.

Despite significant progress, several key questions remain unanswered. Notably, the spatial and temporal regulation of ECM components across HCM stages is poorly defined, and it is unclear how ECM remodeling varies between genotypes or clinical phenotypes. Moreover, the mechanistic links between specific ECM alterations and clinical outcomes—such as arrhythmia burden, progression to heart failure, or sudden cardiac death—are not yet fully elucidated.

To address these gaps, future research should focus on:

• Cell-type-specific and single-cell transcriptomic and proteomic profiling to dissect the heterogeneity of ECM-producing cells and their contributions to fibrosis and hypertrophy.
• Longitudinal and multi-omics studies in HCM patients, integrating advanced imaging, circulating ECM biomarkers, and genetic data to enable more precise phenotyping and outcome prediction.
• Functional validation of ECM-related targets *in vitro* and *in vivo*, using iPSC-based platforms and preclinical animal models to establish causality and therapeutic efficacy.
• Comparative analyses of primary (genetic) versus secondary (acquired) hypertrophy, to delineate shared and divergent ECM remodeling pathways.
• Translational pipelines that link ECM biology to clinical applications—including the development of circulating ECM biomarkers, risk stratification tools, and anti-fibrotic or immunomodulatory therapies.

In conclusion, incorporating ECM biology into the diagnostic, prognostic, and therapeutic frameworks of HCM has the potential to transform patient care. By leveraging innovative *in vitro* disease models and clinically anchored translational research, the field is poised to develop precision-based strategies that address not only the genetic substrate but also the fibrotic and inflammatory landscape that underpins disease progression.

## References

[B1] Allouba M. Walsh R. Afify A. Hosny M. Halawa S. Galal A. (2023). Ethnicity, consanguinity, and genetic architecture of hypertrophic cardiomyopathy. Eur. Heart J. 00, 5146–5158. 10.1093/eurheartj/ehad372 PMC10733735 37431535

[B2] Alter C. Henseler A. S. Owenier C. Hesse J. Ding Z. Lautwein T. (2023). IL-6 in the infarcted heart is preferentially formed by fibroblasts and modulated by purinergic signaling. J. Clin. Invest 133, e163799. 10.1172/JCI163799 36943408 PMC10232006

[B3] Antoniak S. Pawlinski R. Mackman N. (2011). Protease-activated receptors and myocardial infarction. IUBMB Life 63, 383–389. 10.1002/iub.441 21438116 PMC3121912

[B4] Aoyagi T. Matsui T. (2011). The cardiomyocyte as a source of cytokines in cardiac injury. J. Cell Sci. Ther. 2012, 003. 10.4172/2157-7013.s5-003 23493668 PMC3594870

[B5] Argraves W. S. Greene L. M. Cooley M. A. Gallagher W. M. (2003). Fibulins: physiological and disease perspectives. EMBO Rep. 4, 1127–1131. 10.1038/sj.embor.7400033 14647206 PMC1326425

[B6] Barallobre-Barreiro J. Loeys B. Mayr M. Rienks M. Verstraeten A. Kovacic J. C. (2020). Extracellular matrix in vascular disease, part 2/4: JACC focus seminar. J. Am. Coll. Cardiol. 75, 2189–2203. 10.1016/j.jacc.2020.03.018 32354385

[B7] Barallobre-Barreiro J. Radovits T. Fava M. Mayr U. Lin W. Y. Ermolaeva E. (2021). Extracellular matrix in heart failure: role of ADAMTS5 in proteoglycan remodeling. Circulation 144, 2021–2034. 10.1161/CIRCULATIONAHA.121.055732 34806902 PMC8687617

[B8] Bi X. Yang C. Song Y. Yuan J. Cui J. Hu F. (2021). Matrix metalloproteinases increase because of hypoperfusion in obstructive hypertrophic cardiomyopathy. Ann. Thorac. Surg. 111, 915–922. 10.1016/j.athoracsur.2020.05.156 32738221

[B9] Bischoff J. Editor G. Davis G. E. Senger D. R. (2005). Endothelial extracellular matrix. Circ. Res. 97, 1093–1107. 10.1161/01.res.0000191547.64391.e3 16306453

[B10] Brancaccio M. Hirsch E. Notte A. Selvetella G. Lembo G. Tarone G. (2006). Integrin signalling: the tug-of-war in heart hypertrophy. Cardiovasc Res. 70, 422–433. 10.1016/j.cardiores.2005.12.015 16466704

[B11] Brew K. Dinakarpandian D. Nagase H. (2000). Tissue inhibitors of metalloproteinases: evolution, structure and function. Biochim. Biophys. Acta 1477, 267–283. 10.1016/s0167-4838(99)00279-4 10708863

[B12] Brew K. Nagase H. (2010). The tissue inhibitors of metalloproteinases (TIMPs): an ancient family with structural and functional diversity. Biochim. Biophys. Acta 1803, 55–71. 10.1016/j.bbamcr.2010.01.003 20080133 PMC2853873

[B13] Bruggink A. H. van Oosterhout M. F. M. de Jonge N. Cleutjens J. P. M. van Wichen D. F. van Kuik J. (2007). Type IV collagen degradation in the myocardial basement membrane after unloading of the failing heart by a left ventricular assist device. Lab. Investig. 87, 1125–1137. 10.1038/labinvest.3700670 17876299

[B14] Bursac N. (2014). Cardiac fibroblasts in pressure overload hypertrophy: the enemy within? J. Clin. Investigation 124, 2850–2853. 10.1172/JCI76628 PMC4071394 24937423

[B15] Cabral-Pacheco G. A. Garza-Veloz I. Castruita-De la Rosa C. Ramirez-Acuña J. M. Perez-Romero B. A. Guerrero-Rodriguez J. F. (2020). The roles of matrix metalloproteinases and their inhibitors in human diseases. Int. J. Mol. Sci. 21, 9739–9753. 10.3390/ijms21249739 33419373 PMC7767220

[B16] Cambronero F. Marín F. Roldán V. Hernández-Romero D. Valdés M. Lip G. Y. H. (2009). Biomarkers of pathophysiology in hypertrophic cardiomyopathy: implications for clinical management and prognosis. Eur. Heart J. 30, 139–151. 10.1093/eurheartj/ehn538 19136482

[B17] Camelliti P. Borg T. K. Kohl P. (2005). Structural and functional characterisation of cardiac fibroblasts. Cardiovasc Res. 65, 40–51. 10.1016/j.cardiores.2004.08.020 15621032

[B19] Captur G. Heywood W. E. Coats C. Rosmini S. Patel V. Lopes L. R. (2020). Identification of a multiplex biomarker panel for hypertrophic cardiomyopathy using quantitative proteomics and machine learning. Mol. Cell Proteomics 19, 114–127. 10.1074/mcp.RA119.001586 31243064 PMC6944230

[B20] Chait A. den Hartigh L. J. (2020). Adipose tissue distribution, inflammation and its metabolic consequences, including diabetes and cardiovascular disease. Front. Cardiovasc Med. 7, 22. 10.3389/fcvm.2020.00022 32158768 PMC7052117

[B21] Chapman S. L. Sicot F. X. Davis E. C. Huang J. Sasaki T. Chu M. L. (2010). Fibulin-2 and fibulin-5 cooperatively function to form the internal elastic lamina and protect from vascular injury. Arterioscler. Thromb. Vasc. Biol. 30, 68–74. 10.1161/ATVBAHA.109.196725 19893004 PMC2800831

[B22] Chen D. Smith L. R. Khandekar G. Patel P. Yu C. K. Zhang K. (2020). Distinct effects of different matrix proteoglycans on collagen fibrillogenesis and cell-mediated collagen reorganization. Sci. Rep. 10, 19065–13. 10.1038/s41598-020-76107-0 33149218 PMC7642422

[B23] Chen H. Huang X. N. Yan W. Chen K. Guo L. Tummalapali L. (2005). Role of the integrin-linked kinase/PINCH1/alpha-parvin complex in cardiac myocyte hypertrophy. Lab. Investig. 85, 1342–1356. 10.1038/labinvest.3700345 16170337

[B24] Chen R. Zhang H. Tang B. Luo Y. Yang Y. Zhong X. (2024). Macrophages in cardiovascular diseases: molecular mechanisms and therapeutic targets. Signal Transduct. Target. Ther. 9 (1), 130–144. 10.1038/s41392-024-01840-1 38816371 PMC11139930

[B25] Chen X. Q. Li X. Wu X. Ding Y. Li Y. Zhou G. (2023). Integrin beta-like 1 mediates fibroblast–cardiomyocyte crosstalk to promote cardiac fibrosis and hypertrophy. Cardiovasc Res. 119, 1928–1941. 10.1093/cvr/cvad104 37395147

[B26] Chen Y. Wang L. Huang S. Ke J. Wang Q. Zhou Z. (2021). Lutein attenuates angiotensin II- induced cardiac remodeling by inhibiting AP-1/IL-11 signaling. Redox Biol. 44, 102020. 10.1016/j.redox.2021.102020 34077894 PMC8181194

[B27] Chou C. Chin M. T. (2021). Pathogenic mechanisms of hypertrophic cardiomyopathy beyond sarcomere dysfunction. Int. J. Mol. Sci. 22, 8933. 10.3390/ijms22168933 34445638 PMC8396307

[B28] Christensen G. Herum K. M. Lunde I. G. S. (2019). Sweet, yet underappreciated: proteoglycans and extracellular matrix remodeling in heart disease. Matrix Biol. 75–76, 286–299. 10.1016/j.matbio.2018.01.001 29337052

[B29] Chute M. Aujla P. Jana S. Kassiri Z. (2019). The non-fibrillar side of fibrosis: contribution of the basement membrane, Proteoglycans, and glycoproteins to myocardial fibrosis. J. Cardiovasc Dev. Dis. 6, 35. 10.3390/jcdd6040035 31547598 PMC6956278

[B30] Coats C. J. Heywood W. E. Virasami A. Ashrafi N. Syrris P. Dos Remedios C. (2018). Proteomic analysis of the myocardium in hypertrophic obstructive cardiomyopathy. Circ. Genom Precis. Med. 11, e001974. 10.1161/CIRCGEN.117.001974 30562113

[B31] Conte M. Petraglia L. Cabaro S. Valerio V. Poggio P. Pilato E. (2022). Epicardial adipose tissue and cardiac arrhythmias: focus on atrial fibrillation. Front. Cardiovasc Med. 9, 932262. 10.3389/fcvm.2022.932262 35845044 PMC9280076

[B32] Cook S. A. (2023). Understanding interleukin 11 as a disease gene and therapeutic target. Biochem. J. 480, 1987–2008. 10.1042/BCJ20220160 38054591 PMC10754292

[B33] Cooley M. A. Kern C. B. Fresco V. M. Wessels A. Thompson R. P. McQuinn T. C. (2008). Fibulin-1 is required for morphogenesis of neural crest-derived structures. Dev. Biol. 319, 336–345. 10.1016/j.ydbio.2008.04.029 18538758 PMC2965525

[B34] Dabiri B. E. Lee H. Parker K. K. (2012). A potential role for integrin signaling in mechanoelectrical feedback. Prog. Biophys. Mol. Biol. 110, 196–203. 10.1016/j.pbiomolbio.2012.07.002 22819851 PMC4807692

[B35] Davies L. C. Jenkins S. J. Allen J. E. Taylor P. R. (2013). Tissue-resident macrophages. Nat. Immunol. 14, 986–995. 10.1038/ni.2705 24048120 PMC4045180

[B36] Davis G. E. Senger D. R. (2005). Endothelial extracellular matrix: biosynthesis, remodeling, and functions during vascular morphogenesis and neovessel stabilization. Circ. Res. 97, 1093–1107. 10.1161/01.RES.0000191547.64391.e3 16306453

[B37] Diao K. yue Yang Z. G. Xu H. Y. Liu X. Zhang Q. Shi K. (2016). Histologic validation of myocardial fibrosis measured by T1 mapping: a systematic review and meta-analysis. J. Cardiovasc. Magnetic Reson. 18, 92–11. 10.1186/s12968-016-0313-7 PMC5154013 27955698

[B38] Díez J. González A. Kovacic J. C. (2020). Myocardial interstitial fibrosis in nonischemic heart disease, part 3/4: JACC focus seminar. J. Am. Coll. Cardiol. 75, 2204–2218. 10.1016/j.jacc.2020.03.019 32354386 PMC7213023

[B39] Dobaczewski M. Chen W. Frangogiannis N. G. (2011). Transforming growth factor (TGF)-β signaling in cardiac remodeling. J. Mol. Cell Cardiol. 51, 600–606. 10.1016/j.yjmcc.2010.10.033 21059352 PMC3072437

[B40] Dour C.Le Wu W. Béréziat V. Capeau J. Vigouroux C. Worman H. J. (2017). Extracellular matrix remodeling and transforming growth factor-βsignaling abnormalities induced by lamin a/c variants that cause lipodystrophy. J. Lipid Res. 58, 151–163. 10.1194/jlr.M071381 27845687 PMC5234718

[B41] Eghbali M. Weber K. T. (1990). Collagen and the myocardium: fibrillar structure, biosynthesis and degradation in relation to hypertrophy and its regression. Mol. Cell Biochem. 96, 1–14. 10.1007/BF00228448 2146489

[B42] Ellims A. H. Taylor A. J. Mariani J. A. Ling L. H. Iles L. M. Maeder M. T. (2014). Evaluating the utility of circulating biomarkers of collagen synthesis in hypertrophic cardiomyopathy. Circ. Heart Fail 7, 271–278. 10.1161/CIRCHEARTFAILURE.113.000665 24481111

[B43] Espeland T. Lunde I. G. Amundsen B. H. Gullestad L. Aakhus S. (2018). Espeland and co-workers respond. Tidsskrift Den norske legeforening 138. 10.4045/tidsskr.18.0865 30497251

[B44] Fan D. Takawale A. Lee J. Kassiri Z. (2012). Cardiac fibroblasts, fibrosis and extracellular matrix remodeling in heart disease. Fibrogenesis and Tissue Repair 2012 5 (1), 15–13. 10.1186/1755-1536-5-15 PMC3464725 22943504

[B45] Fang L. Ellims A. H. Beale A. L. Taylor A. J. Murphy A. Dart A. M. (2017). Systemic inflammation is associated with myocardial fibrosis, diastolic dysfunction, and cardiac hypertrophy in patients with hypertrophic cardiomyopathy. Am. J. Transl. Res. 9, 5063–5073. 29218105 PMC5714791

[B46] Farhadian F. Contard F. Sabri A. Samuel J. L. Rappaport L. (1996). Fibronectin and basement membrane in cardiovascular organogenesis and disease pathogenesis. Cardiovasc Res. 32, 433–442. 10.1016/s0008-6363(96)00119-8 8881506

[B47] Feldman A. M. Combes A. Wagner D. Kadakomi T. Kubota T. Li Y. Y. (2000). The role of tumor necrosis factor in the pathophysiology of heart failure. J. Am. Coll. Cardiol. 35, 537–544. 10.1016/s0735-1097(99)00600-2 10716453

[B48] Fernlund E. Gyllenhammar T. Jablonowski R. Carlsson M. Larsson A. Ärnlöv J. (2017). Serum biomarkers of myocardial remodeling and coronary dysfunction in early stages of hypertrophic cardiomyopathy in the young. Pediatr. Cardiol. 38, 853–863. 10.1007/s00246-017-1593-x 28361263 PMC5388706

[B49] Finsen A. V. Lunde I. G. Sjaastad I. Østli E. K. Lyngra M. Jarstadmarken H. O. (2011). Syndecan-4 is essential for development of concentric myocardial hypertrophy *via* stretch-induced activation of the Calcineurin-NFAT pathway. PLoS One 6, e28302. 10.1371/journal.pone.0028302 22164265 PMC3229559

[B50] Foronjy R. F. Sun J. Lemaitre V. d’Armiento J. M. (2008). Transgenic expression of matrix Metalloproteinase-1 inhibits myocardial fibrosis and prevents the transition to heart failure in a pressure overload mouse model. Hypertens. Res. 31 (4), 725–735. 10.1291/hypres.31.725 18633185

[B51] Frangogiannis N. G. (2020). Transforming growth Factor–ß in tissue fibrosis. J. Exp. Med. 217, e20190103. 10.1084/jem.20190103 32997468 PMC7062524

[B52] Frieler R. A. Mortensen R. M. (2015). Immune cell and other non-cardiomyocyte regulation of cardiac hypertrophy and remodeling. Circulation 131, 1019–1030. 10.1161/CIRCULATIONAHA.114.008788 25779542 PMC4367123

[B53] Früh S. M. Schoen I. Ries J. Vogel V. (2015). Molecular architecture of native fibronectin fibrils. Nat. Commun. 6, 7275–10. 10.1038/ncomms8275 26041410 PMC4468872

[B54] Fucikova A. Lenco J. Tambor V. Rehulkova H. Pudil R. Stulik J. (2016). Plasma concentration of fibronectin is decreased in patients with hypertrophic cardiomyopathy. Clin. Chim. Acta 463, 62–66. 10.1016/j.cca.2016.09.024 27693530

[B55] Fujiu K. Nagai R. (2014). Fibroblast-mediated pathways in cardiac hypertrophy. J. Mol. Cell Cardiol. 70, 64–73. 10.1016/j.yjmcc.2014.01.013 24492068

[B56] Gogiraju R. Bochenek M. L. Schäfer K. (2019). Angiogenic endothelial cell signaling in cardiac hypertrophy and heart failure. Front. Cardiovasc Med. 6, 20. 10.3389/fcvm.2019.00020 30895179 PMC6415587

[B57] Groot H. E. Al Ali L. van der Horst I. C. C. Schurer R. A. J. van der Werf H. W. Lipsic E. (2019). Plasma interleukin 6 levels are associated with cardiac function after ST-elevation myocardial infarction. Clin. Res. Cardiol. 108, 612–621. 10.1007/s00392-018-1387-z 30367209 PMC6529378

[B58] Guo J. Wang Y. Liang H. Yang B. (2023). Small leucine rich proteoglycan in fibrotic diseases: new frenemies? Int. J. Drug Discov. Pharmacol. 61–78, 61–78. 10.53941/IJDDP.2023.100005

[B59] Haaf P. Garg P. Messroghli D. R. Broadbent D. A. Greenwood J. P. Plein S. (2016). Cardiac T1 mapping and extracellular volume (ECV) in clinical practice: a comprehensive review. J. Cardiovasc. Magnetic Reson. 18, 89–12. 10.1186/s12968-016-0308-4 PMC5129251 27899132

[B60] Hajsadeghi F. Nabavi V. Bhandari A. Choi A. Vincent H. Flores F. (2014). Increased epicardial adipose tissue is associated with coronary artery disease and major adverse cardiovascular events. Atherosclerosis 237, 486–489. 10.1016/j.atherosclerosis.2014.09.037 25463078

[B61] Halabi C. M. Broekelmann T. J. Lin M. Lee V. S. Chu M. L. Mecham R. P. (2017). Fibulin-4 is essential for maintaining arterial wall integrity in conduit but not muscular arteries. Sci. Adv. 3, e1602532. 10.1126/sciadv.1602532 28508064 PMC5415335

[B62] Hall C. Gehmlich K. Denning C. Pavlovic D. (2021). Complex relationship between cardiac fibroblasts and cardiomyocytes in health and disease. J. Am. Heart Assoc. 10, e019338–15. 10.1161/JAHA.120.019338 33586463 PMC8174279

[B63] Hanna A. Frangogiannis N. G. (2019). The role of the TGF-β superfamily in myocardial infarction. Front. Cardiovasc Med. 6, 140. 10.3389/fcvm.2019.00140 31620450 PMC6760019

[B64] Hanna A. Frangogiannis N. G. (2020). Inflammatory cytokines and chemokines as therapeutic targets in heart failure. Cardiovasc Drugs Ther. 34, 849–863. 10.1007/s10557-020-07071-0 32902739 PMC7479403

[B65] Harston R. K. Kuppuswamy D. (2011). Integrins are the necessary links to hypertrophic growth in cardiomyocytes. J. Signal Transduct. 2011, 521742–521748. 10.1155/2011/521742 21637377 PMC3101892

[B66] Hasegawa K. Fujiwara H. Doyama K. Miyamae M. Fujiwara T. Suga S. (1993). Ventricular expression of brain natriuretic peptide in hypertrophic cardiomyopathy. Circulation 88, 372–380. 10.1161/01.cir.88.2.372 8339400

[B67] Heiss C. Rodriguez-Mateos A. Kelm M. (2015). Central role of eNOS in the maintenance of endothelial homeostasis. Antioxid. Redox Signal 22, 1230–1242. 10.1089/ars.2014.6158 25330054 PMC4410282

[B68] Herum K. M. Lunde I. G. Skrbic B. Louch W. E. Hasic A. Boye S. (2015). Syndecan-4 is a key determinant of collagen cross-linking and passive myocardial stiffness in the pressure-overloaded heart. Cardiovasc Res. 106, 217–226. 10.1093/cvr/cvv002 25587045

[B69] Herum K. M. Romaine A. Wang A. Melleby A. O. Strand M. E. Pacheco J. (2020). Syndecan-4 protects the heart from the profibrotic effects of thrombin-cleaved osteopontin. J. Am. Heart Assoc. 9, e013518. 10.1161/JAHA.119.013518 32000579 PMC7033859

[B70] Hitscherich P. Lee E. J. (2021). Crosstalk between cardiac cells and macrophages postmyocardial infarction: insights from *in vitro* studies. Tissue Eng. Part B Rev. 27, 475–485. 10.1089/ten.TEB.2020.0198 33096955 PMC8851218

[B71] Ho C. Y. López B. Coelho-Filho O. R. Lakdawala N. K. Cirino A. L. Jarolim P. (2010). Myocardial fibrosis as an early manifestation of hypertrophic cardiomyopathy. N. Engl. J. Med. 363, 552–563. 10.1056/NEJMoa1002659 20818890 PMC3049917

[B18] Ho J. E. Shi L. Day S. M. Colan S. D. Russell M. W. Towbin J. A. (2017). Biomarkers of cardiovascular stress and fibrosis in preclinical hypertrophic cardiomyopathy. Open Heart 4 (2), e000615. 10.1136/openhrt-2017-000615 29177058 PMC5687543

[B72] Högye M. Mándi Y. Csanády M. Sepp R. Buzás K. (2004). Comparison of circulating levels of interleukin-6 and tumor necrosis factor-alpha in hypertrophic cardiomyopathy and in idiopathic dilated cardiomyopathy. Am. J. Cardiol. 94, 249–251. 10.1016/j.amjcard.2004.03.078 15246916

[B73] Hotta Y. Sasaki S. Konishi M. Kinoshita H. Kuwahara K. Nakao K. (2008). Fgf16 is required for cardiomyocyte proliferation in the mouse embryonic heart. Dev. Dyn. 237, 2947–2954. 10.1002/dvdy.21726 18816849

[B74] Hultgårdh-Nilsson A. Borén J. Chakravarti S. (2015). The small leucine-rich repeat Proteoglycans in tissue repair and atherosclerosis. J. Intern Med. 278, 447–461. 10.1111/joim.12400 26477596 PMC4616156

[B75] Hynes R. O. Naba A. (2012). Overview of the matrisome—an inventory of extracellular matrix constituents and functions. Cold Spring Harb. Perspect. Biol. 4, a004903. 10.1101/cshperspect.a004903 21937732 PMC3249625

[B76] Ibrahim A. M. Galal A. Halawa S. Elfawy H. Elshorbagy S. Roshdy M. (2022a). Abstract 14969: transcriptome signature of cardiac fibroblasts in HCM patients identifies novel drivers of ECM remodeling and pro-inflammatory signaling. Circulation 146. 10.1161/circ.146.suppl_1.14969

[B77] Ibrahim A. M. Moss M. A. Gray Z. Rojo M. D. Burke C. M. Schwertfeger K. L. (2020b). Diverse macrophage populations contribute to the inflammatory microenvironment in premalignant lesions during localized invasion. Front. Oncol. 10, 569985. 10.3389/fonc.2020.569985 33072601 PMC7541939

[B78] Ibrahim A. M. Roshdy M. Elshorbagy S. Hosny M. Halawa S. Yehia D. (2020a). An investigation of Fibulin-2 in hypertrophic cardiomyopathy. Int. J. Mol. Sci. 21, 1–14. 10.3390/ijms21197176 PMC7583916 33003281

[B79] Ibrahim A. M. Roshdy M. Latif N. Sarathchandra P. Hosny M. Haikal S. (2022b). Structural, molecular and functional characterization of the aorta in HCM. Eur. Heart J. 43. 10.1093/eurheartj/ehac544.1935

[B80] Ibrahim A. M. Sabet S. El-Ghor A. A. Kamel N. Anis S. E. Morris J. S. (2018). Fibulin-2 is required for basement membrane integrity of mammary epithelium. Sci. Rep. 8, 14139. 10.1038/s41598-018-32507-x 30237579 PMC6148073

[B81] Imanaka-Yoshida K. Hiroe M. Nishikawa T. Ishiyama S. Shimojo T. Ohta Y. (2001). Tenascin-C modulates adhesion of cardiomyocytes to extracellular matrix during tissue remodeling after myocardial infarction. Lab. Investig. 81, 1015–1024. 10.1038/labinvest.3780313 11454990

[B82] Imanaka-Yoshida K. Tawara I. Yoshida T. (2020). Tenascin-C in cardiac disease: a sophisticated controller of inflammation, repair, and fibrosis. Am. J. Physiol. Cell Physiol. 319, C781-C796–C796. 10.1152/ajpcell.00353.2020 32845719

[B83] Ioakeimidis N. S. Pitsis A. Zegkos T. Ntelios D. Kelpis T. Papamitsou T. (2023). Periostin is overexpressed, correlated with fibrosis and differs among grades of cardiomyocyte hypertrophy in myectomy tissue of patients with hypertrophic cardiomyopathy. PLoS One 18, e0293427. 10.1371/journal.pone.0293427 37939043 PMC10631645

[B84] Israeli-Rosenberg S. Manso A. M. Okada H. Ross R. S. (2014). Integrins and integrin-associated proteins in the cardiac myocyte. Circ. Res. 114, 572–586. 10.1161/CIRCRESAHA.114.301275 24481847 PMC3975046

[B85] Itoh N. Ohta H. (2013). Pathophysiological roles of FGF signaling in the heart. Front. Physiol. 4 (SEP), 247. 10.3389/fphys.2013.00247 24046748 PMC3764331

[B86] Itoh N. Ornitz D. M. (2008). Functional evolutionary history of the mouse fgf gene family. Dev. Dyn. 237, 18–27. 10.1002/dvdy.21388 18058912

[B87] Iwasaki J. Nakamura K. Matsubara H. Nakamura Y. Nishii N. Banba K. (2009). Relationship between circulating levels of monocyte chemoattractant protein-1 and systolic dysfunction in patients with hypertrophic cardiomyopathy. Cardiovasc. Pathol. 18, 317–322. 10.1016/j.carpath.2008.12.004 19211266

[B88] Jahanyar J. Joyce D. L. Southard R. E. Loebe M. Noon G. P. Koerner M. M. (2007). Decorin-mediated transforming growth Factor-β inhibition ameliorates adverse cardiac remodeling. J. Heart Lung Transplant. 26, 34–40. 10.1016/j.healun.2006.10.005 17234515

[B89] Jallerat Q. Feinberg A. W. (2020). Extracellular matrix structure and composition in the early four-chambered embryonic heart. Cells 9, 285. 10.3390/cells9020285 31991580 PMC7072588

[B90] Jang D. I. Lee A. H. Shin H. Y. Song H. R. Park J. H. Kang T. B. (2021). The role of tumor necrosis factor alpha (TNF-α) in autoimmune disease and current TNF-α inhibitors in therapeutics. Int. J. Mol. Sci. 22, 2719. 10.3390/ijms22052719 33800290 PMC7962638

[B91] Jebran A.-F. Seidler T. Tiburcy M. Daskalaki M. Kutschka I. Fujita B. (2025). Engineered heart muscle allografts for heart repair in Primates and humans. Nature 639 (8054), 503–511. 10.1038/s41586-024-08463-0 39880949 PMC11903342

[B92] Jobe L. J. Meléndez G. C. Levick S. P. Du Y. Brower G. L. Janicki J. S. (2009). TNF-α inhibition attenuates adverse myocardial remodeling in a rat model of volume overload. Am. J. Physiol. Heart Circ. Physiol. 297, 1462–1468. 10.1152/ajpheart.00442.2009 PMC2770768 19666842

[B93] Johnson B. B. Cosson M. V. Tsansizi L. I. Holmes T. L. Gilmore T. Hampton K. (2024). Perlecan (HSPG2) promotes structural, contractile, and metabolic development of human cardiomyocytes. Cell Rep. 43, 113668. 10.1016/j.celrep.2023.113668 38198277

[B94] Kanisicak O. Khalil H. Ivey M. J. Karch J. Maliken B. D. Correll R. N. (2016). Genetic lineage tracing defines myofibroblast origin and function in the injured heart. Nat. Commun. 7, 12260. 10.1038/ncomms12260 27447449 PMC5512625

[B95] Khan S. A. Dong H. Joyce J. Sasaki T. Chu M. L. Tsuda T. (2016). Fibulin-2 is essential for angiotensin II-induced myocardial fibrosis mediated by transforming growth factor (TGF)-β. Lab. Invest 96, 773–783. 10.1038/labinvest.2016.52 27111286 PMC4920723

[B96] Kitaoka H. Kubo T. Baba Y. Yamasaki N. Matsumura Y. Furuno T. (2012). Serum tenascin-C levels as a prognostic biomarker of heart failure events in patients with hypertrophic cardiomyopathy. J. Cardiol. 59, 209–214. 10.1016/j.jjcc.2011.11.008 22218323

[B97] Kitaoka H. Kubo T. Okawa M. Hayato K. Yamasaki N. Matsumura Y. (2010). Impact of metalloproteinases on left ventricular remodeling and heart failure events in patients with hypertrophic cardiomyopathy. Circulation J. 74, 1191–1196. 10.1253/circj.cj-09-1013 20453389

[B98] Kitaoka H. Kubo T. Okawa M. Takenaka N. Baba Y. Yamasaki N. (2011). Plasma metalloproteinase levels and left ventricular remodeling in hypertrophic cardiomyopathy in patients with an identical mutation. J. Cardiol. 58, 261–265. 10.1016/j.jjcc.2011.07.011 21890325

[B99] Koch C. D. Lee C. M. Apte S. S. (2020). Aggrecan in cardiovascular development and disease. J. Histochem Cytochem 68, 777–795. 10.1369/0022155420952902 32870742 PMC7649964

[B100] Konstandin M. H. Völkers M. Collins B. Quijada P. Quintana M. De La Torre A. (2013). Fibronectin contributes to pathological cardiac hypertrophy but not physiological growth. Basic Res. Cardiol. 108, 375. 10.1007/s00395-013-0375-8 23912225 PMC3813434

[B101] Krawetz R. J. Wu Y. E. Bertram K. L. Shonak A. Masson A. O. Ren G. (2022). Synovial mesenchymal progenitor derived aggrecan regulates cartilage homeostasis and endogenous repair capacity. Cell Death and Dis. 13 (5), 470–16. 10.1038/s41419-022-04919-1 PMC9117284 35585042

[B102] Krishnan A. Chilton E. Raman J. Saxena P. McFarlane C. Trollope A. F. (2021). Are interactions between epicardial adipose tissue, cardiac fibroblasts and cardiac myocytes instrumental in atrial fibrosis and atrial fibrillation? Cells 10, 2501. 10.3390/cells10092501 34572150 PMC8467050

[B103] Kühn B. del Monte F. Hajjar R. J. Chang Y. S. Lebeche D. Arab S. (2007). Periostin induces proliferation of differentiated cardiomyocytes and promotes cardiac repair. Nat. Med. 13 (8), 962–969. 10.1038/nm1619 17632525

[B104] Lasala G. P. Silva J. A. Allers C. Minguell J. J. (2012). Combination cell therapy for the treatment of acute myocardial infarction. Int. J. Cardiol. 157, 293–294. 10.1016/j.ijcard.2012.03.125 22483420

[B105] Laser M. Willey C. D. Jiang W. Cooper G. 4th Menick D. R. Zile M. R. (2000). Integrin activation and focal complex formation in cardiac hypertrophy. J. Biol. Chem. 275, 35624–35630. 10.1074/jbc.M006124200 10958798

[B106] Lavine K. J. Pinto A. R. Epelman S. Kopecky B. J. Clemente-Casares X. Godwin J. (2018). The macrophage in cardiac homeostasis and disease: JACC macrophage in CVD series (part 4). J. Am. Coll. Cardiol. 72, 2213–2230. 10.1016/j.jacc.2018.08.2149 30360829 PMC6209119

[B107] Li G. Xie J. Chen J. Li R. Wu H. Zhang X. (2017). Syndecan-4 deficiency accelerates the transition from compensated hypertrophy to heart failure following pressure overload. Cardiovasc. Pathol. 28, 74–79. 10.1016/j.carpath.2017.03.008 28395201

[B108] Li L. Fan D. Wang C. Wang J. Y. Cui X. B. Wu D. (2011). Angiotensin II increases periostin expression *via* Ras/p38 MAPK/CREB and ERK1/2/TGF-β1 pathways in cardiac fibroblasts. Cardiovasc Res. 91, 80–89. 10.1093/cvr/cvr067 21367774

[B109] Lillo R. Graziani F. Franceschi F. Iannaccone G. Massetti M. Olivotto I. (2023). Inflammation across the spectrum of hypertrophic cardiac phenotypes. Heart Fail Rev. 28, 1065–1075. 10.1007/s10741-023-10307-4 37115472 PMC10403403

[B110] Lim D. S. Lutucuta S. Bachireddy P. Youker K. Evans A. Entman M. (2001). Angiotensin II blockade reverses myocardial fibrosis in a transgenic mouse model of human hypertrophic cardiomyopathy. Circulation 103, 789–791. 10.1161/01.cir.103.6.789 11171784 PMC2779524

[B111] Loeys B. L. Chen J. Neptune E. R. Judge D. P. Podowski M. Holm T. (2005). A syndrome of altered cardiovascular, craniofacial, neurocognitive and skeletal development caused by mutations in TGFBR1 or TGFBR2. Nat. Genet. 37 (3), 275–281. 10.1038/ng1511 15731757

[B112] Lombardi R. Betocchi S. Losi M. A. Tocchetti C. G. Aversa M. Miranda M. (2003). Myocardial collagen turnover in hypertrophic cardiomyopathy. Circulation 108, 1455–1460. 10.1161/01.CIR.0000090687.97972.10 12952838

[B113] Lunde I. G. Aronsen J. M. Melleby A. O. Strand M. E. Skogestad J. Bendiksen B. A. (2022). Cardiomyocyte-specific overexpression of syndecan-4 in mice results in activation of calcineurin-NFAT signalling and exacerbated cardiac hypertrophy. Mol. Biol. Rep. 49, 11795–11809. 10.1007/s11033-022-07985-y 36205855 PMC9712407

[B114] Lunde I. G. Herum K. M. Carlson C. C. Christensen G. (2016). Syndecans in heart fibrosis. Cell Tissue Res. 365, 539–552. 10.1007/s00441-016-2454-2 27411689

[B115] Macintyre C. Lakdawala N. K. (2016). Management of atrial fibrillation in hypertrophic cardiomyopathy. Circulation 133, 1901–1905. 10.1161/CIRCULATIONAHA.115.015085 27166348

[B116] Mai J. Virtue A. Shen J. Wang H. Yang X. F. (2013). An evolving new paradigm: endothelial cells – conditional innate immune cells. J. Hematol. and Oncol. 6 (1), 61–13. 10.1186/1756-8722-6-61 23965413 PMC3765446

[B117] Maitra N. Flink I. L. Bahl J. J. Morkin E. (2000). Expression of alpha and beta integrins during terminal differentiation of cardiomyocytes. Cardiovasc Res. 47, 715–725. 10.1016/s0008-6363(00)00140-1 10974220

[B118] Mann D. L. (2015). Innate immunity and the failing heart: the cytokine hypothesis revisited. Circ. Res. 116, 1254–1268. 10.1161/CIRCRESAHA.116.302317 25814686 PMC4380242

[B119] Marian A. J. Braunwald E. (2017). Hypertrophic cardiomyopathy: genetics, pathogenesis, clinical manifestations, diagnosis, and therapy. Circ. Res. 121, 749–770. 10.1161/CIRCRESAHA.117.311059 28912181 PMC5654557

[B120] Maron B. J. (2002). Hypertrophic cardiomyopathy: a systematic review. JAMA 287, 1308–1320. 10.1001/jama.287.10.1308 11886323

[B121] Martino F. Perestrelo A. R. Vinarský V. Pagliari S. Forte G. (2018). Cellular mechanotransduction: from tension to function. Front. Physiol. 9, 824. 10.3389/fphys.2018.00824 30026699 PMC6041413

[B122] Marwick T. H. Narula J. (2010). Cardiac ultrasound imaging in acute care settings. JACC Cardiovasc Imaging 3, 671–672. 10.1016/j.jcmg.2010.04.005 20541726

[B123] Mathiesen S. B. Lunde M. Aronsen J. M. Romaine A. Kaupang A. Martinsen M. (2019). The cardiac syndecan-4 interactome reveals a role for syndecan-4 in nuclear translocation of muscle LIM protein (MLP). J. Biol. Chem. 294, 8717–8731. 10.1074/jbc.RA118.006423 30967474 PMC6552415

[B124] Matsui Y. Jia N. Okamoto H. Kon S. Onozuka H. Akino M. (2004). Role of osteopontin in cardiac fibrosis and remodeling in angiotensin II-Induced cardiac hypertrophy. Hypertension 43, 1195–1201. 10.1161/01.HYP.0000128621.68160.dd 15123578

[B125] Matsumoto E. Sasaki S. Kinoshita H. Kito T. Ohta H. Konishi M. (2013). Angiotensin II-induced cardiac hypertrophy and fibrosis are promoted in mice lacking Fgf16. Genes Cells 18, 544–553. 10.1111/gtc.12055 23600527 PMC3738920

[B126] Matthia E. L. Setteducato M. L. Elzeneini M. Vernace N. Salerno M. Kramer C. M. (2022). Circulating biomarkers in hypertrophic cardiomyopathy. J. Am. Heart Assoc. 11, e027618. 10.1161/JAHA.122.027618 36382968 PMC9851432

[B127] McLaughlin P. J. Bakall B. Choi J. Liu Z. Sasaki T. Davis E. C. (2007). Lack of fibulin-3 causes early aging and herniation, but not macular degeneration in mice. Hum. Mol. Genet. 16, 3059–3070. 10.1093/hmg/ddm264 17872905

[B128] Meléndez G. C. McLarty J. L. Levick S. P. Du Y. Janicki J. S. Brower G. L. (2010). Interleukin-6 mediates myocardial fibrosis, concentric hypertrophy and diastolic dysfunction in rats. Hypertension 56, 225–231. 10.1161/HYPERTENSIONAHA.109.148635 20606113 PMC2921860

[B129] Merline R. Schaefer R. M. Schaefer L. (2009). The matricellular functions of small leucine-rich proteoglycans (SLRPs). J. Cell Commun. Signal 3, 323–335. 10.1007/s12079-009-0066-2 19809894 PMC2778586

[B130] Mezu-Ndubuisi O. J. Maheshwari A. (2020). The role of integrins in inflammation and angiogenesis. Pediatr. Res. 89 (7), 1619–1626. 10.1038/s41390-020-01177-9 33027803 PMC8249239

[B131] Miao K. Zhou L. Ba H. Li C. Gu H. Yin B. (2020). Transmembrane tumor necrosis factor alpha attenuates pressure-overload cardiac hypertrophy *via* tumor necrosis factor receptor 2. PLoS Biol. 18, e3000967. 10.1371/journal.pbio.3000967 33270628 PMC7714153

[B132] Mohamed I. A. Gadeau A. P. Hasan A. Abdulrahman N. Mraiche F. O. (2019). Promising therapeutic target in cardiac fibrosis. Cells 8. 10.3390/cells8121558 PMC6952988 31816901

[B133] Mohammadzadeh N. Lunde I. G. Andenæs K. Strand M. E. Aronsen J. M. Skrbic B. (2019). The extracellular matrix proteoglycan lumican improves survival and counteracts cardiac dilatation and failure in mice subjected to pressure overload. Sci. Rep. 9, 9206. 10.1038/s41598-019-45651-9 31235849 PMC6591256

[B134] Mohammadzadeh N. Melleby A. O. Palmero S. Sjaastad I. Chakravarti S. Engebretsen K. V. T. (2020). Moderate loss of the extracellular matrix proteoglycan lumican attenuates cardiac fibrosis in mice subjected to pressure overload. Cardiology 145, 187–198. 10.1159/000505318 31968347 PMC7672712

[B135] Moretti F. A. Chauhan A. K. Iaconcig A. Porro F. Baralle F. E. Muro A. F. (2007). A major fraction of fibronectin present in the extracellular matrix of tissues is plasma-derived. J. Biol. Chem. 282, 28057–28062. 10.1074/jbc.M611315200 17644525

[B136] Morimoto H. Takahashi M. Izawa A. Ise H. Hongo M. Kolattukudy P. E. (2006). Cardiac overexpression of monocyte chemoattractant Protein-1 in transgenic mice prevents cardiac dysfunction and remodeling after myocardial infarction. Circ. Res. 99, 891–899. 10.1161/01.RES.0000246113.82111.2d 16990567

[B137] Mouw J. K. Ou G. Weaver V. M. (2014). Extracellular matrix assembly: a multiscale deconstruction. Nat. Rev. Mol. Cell Biol. 15, 771–785. 10.1038/nrm3902 25370693 PMC4682873

[B138] Naba A. Clauser K. R. Hoersch S. Liu H. Carr S. A. Hynes R. O. (2012). The matrisome: *in silico* definition and *in vivo* characterization by proteomics of normal and tumor extracellular matrices. Mol. Cell. Proteomics 11, M111.014647. 10.1074/mcp.M111.014647 PMC3322572 22159717

[B139] Nagase H. Visse R. Murphy G. (2006). Structure and function of matrix metalloproteinases and TIMPs. Cardiovasc Res. 69, 562–573. 10.1016/j.cardiores.2005.12.002 16405877

[B140] Nakamura Y. Kita S. Tanaka Y. Fukuda S. Obata Y. Okita T. (2020). A disintegrin and metalloproteinase 12 prevents heart failure by regulating cardiac hypertrophy and fibrosis. Am. J. Physiol. Heart Circ. Physiol. 318, H238-H251–H251. 10.1152/ajpheart.00496.2019 31774689

[B141] Nakasaki M. Hwang Y. Xie Y. Kataria S. Gund R. Hajam E. Y. (2015). The matrix protein Fibulin-5 is at the interface of tissue stiffness and inflammation in fibrosis. Nat. Commun. 6, 8574. 10.1038/ncomms9574 26469761 PMC4634219

[B142] Nikitovic D. Katonis P. Tsatsakis A. Karamanos N. K. Tzanakakis G. N. (2008). Lumican, a small leucine-rich proteoglycan. IUBMB Life 60, 818–823. 10.1002/iub.131 18949819

[B143] Nikolov A. Popovski N. (2022). Extracellular matrix in heart disease: focus on circulating collagen type I and III derived peptides as biomarkers of myocardial fibrosis and their potential in the prognosis of heart failure: a concise review. Metabolites 12, 297. 10.3390/metabo12040297 35448484 PMC9025448

[B144] Norris R. A. Moreno-Rodriguez R. A. Sugi Y. Hoffman S. Amos J. Hart M. M. (2008). Periostin regulates atrioventricular valve maturation. Dev. Biol. 316, 200–213. 10.1016/j.ydbio.2008.01.003 18313657 PMC2386672

[B145] Norris R. A. Potts J. D. Yost M. J. Junor L. Brooks T. Tan H. (2009). Periostin promotes a fibroblastic lineage pathway in atrioventricular valve progenitor cells. Dev. Dyn. 238, 1052–1063. 10.1002/dvdy.21933 19334280 PMC2886283

[B146] Ogawa E. Saito Y. Kuwahara K. Harada M. Miyamoto Y. Hamanaka I. (2002). Fibronectin signaling stimulates BNP gene transcription by inhibiting neuron-restrictive silencer element-dependent repression. Cardiovasc Res. 53, 451–459. 10.1016/s0008-6363(01)00492-8 11827696

[B147] O’Hanlon R. Grasso A. Roughton M. Moon J. C. Clark S. Wage R. (2010). Prognostic significance of myocardial fibrosis in hypertrophic cardiomyopathy. J. Am. Coll. Cardiol. 56, 867–874. 10.1016/j.jacc.2010.05.010 20688032

[B148] Olijnyk D. Ibrahim A. M. Ferrier R. K. Tsuda T. Chu M. L. Gusterson B. A. (2014). Fibulin-2 is involved in early extracellular matrix development of the outgrowing mouse mammary epithelium. Cell Mol. Life Sci. 71, 3811–3828. 10.1007/s00018-014-1577-4 24522256 PMC11113845

[B149] Oliviéro P. Chassagne C. Salichon N. Corbier A. Hamon G. Marotte F. (2000). Expression of laminin alpha2 chain during normal and pathological growth of myocardium in rat and human. Cardiovasc Res. 46, 346–355. 10.1016/s0008-6363(00)00034-1 10773239

[B150] Olivotto I. Maron M. S. Autore C. Lesser J. R. Rega L. Casolo G. (2008). Assessment and significance of left ventricular mass by cardiovascular magnetic resonance in hypertrophic cardiomyopathy. J. Am. Coll. Cardiol. 52, 559–566. 10.1016/j.jacc.2008.04.047 18687251

[B151] O’Rourke S. A. Dunne A. Monaghan M. G. (2019). The role of macrophages in the infarcted myocardium: orchestrators of ECM remodeling. Front. Cardiovasc Med. 6, 101. 10.3389/fcvm.2019.00101 31417911 PMC6685361

[B152] Piek A. de Boer R. A. Silljé H. H. W. (2016). The fibrosis-cell death axis in heart failure. Heart Fail. Rev. 21 (2), 199–211. 10.1007/s10741-016-9536-9 26883434 PMC4762920

[B153] Piers S. R. D. van Huls van Taxis C. F. B. Tao Q. van der Geest R. J. Askar S. F. Siebelink H. M. J. (2013). Epicardial substrate mapping for ventricular tachycardia ablation in patients with non-ischaemic cardiomyopathy: a new algorithm to differentiate between scar and viable myocardium developed by simultaneous integration of computed tomography and contrast-enhanced magnetic resonance imaging. Eur. Heart J. 34, 586–596. 10.1093/eurheartj/ehs382 23161702

[B154] Podesser B. K. Kreibich M. Dzilic E. Santer D. Förster L. Trojanek S. (2018). Tenascin-C promotes chronic pressure overload-induced cardiac dysfunction, hypertrophy and myocardial fibrosis. J. Hypertens. 36, 847–856. 10.1097/HJH.0000000000001628 29283973

[B155] Previs M. J. O'Leary T. S. Morley M. P. Palmer B. M. LeWinter M. Yob J. M. (2022). Defects in the proteome and metabolome in human hypertrophic cardiomyopathy. Circ. Heart Fail 15, E009521. 10.1161/CIRCHEARTFAILURE.121.009521 35543134 PMC9708114

[B156] Repetti G. G. Kim Y. Pereira A. C. Ingles J. Russell M. W. Lakdawala N. K. (2021). Discordant clinical features of identical hypertrophic cardiomyopathy twins. Proc. Natl. Acad. Sci. U. S. A. 118, e2021717118. 10.1073/pnas.2021717118 33658374 PMC7958207

[B157] Revelo X. S. Parthiban P. Chen C. Barrow F. Fredrickson G. Wang H. (2021). Cardiac resident macrophages prevent fibrosis and stimulate angiogenesis. Circ. Res. 129, 1086–1101. 10.1161/CIRCRESAHA.121.319737 34645281 PMC8638822

[B158] Ricard-Blum S. (2011). The collagen family. Cold Spring Harb. Perspect. Biol. 3, a004978–19. 10.1101/cshperspect.a004978 21421911 PMC3003457

[B159] Rienks M. Papageorgiou A. P. Frangogiannis N. G. Heymans S. (2014). Myocardial extracellular matrix: an ever-changing and diverse entity. Circ. Res. 114, 872–888. 10.1161/CIRCRESAHA.114.302533 24577967

[B160] Rixon C. Andreassen K. Shen X. Erusappan P. M. Almaas V. M. Palmero S. (2023). Lumican accumulates with fibrillar collagen in fibrosis in hypertrophic cardiomyopathy. Esc. Heart Fail 10, 858–871. 10.1002/ehf2.14234 36444917 PMC10053290

[B161] Rodríguez D. Morrison C. J. Overall C. M. (2010). Matrix metalloproteinases: what do they not do? New substrates and biological roles identified by murine models and proteomics. Biochim. Biophys. Acta 1803, 39–54. 10.1016/j.bbamcr.2009.09.015 19800373

[B162] Roldán V. Marín F. Gimeno J. R. Ruiz-Espejo F. González J. Feliu E. (2008). Matrix metalloproteinases and tissue remodeling in hypertrophic cardiomyopathy. Am. Heart J. 156, 85–91. 10.1016/j.ahj.2008.01.035 18585501

[B163] Ross R. S. (2002). The extracellular connections: the role of integrins in myocardial remodeling. J. Card. Fail 8, S326–S331. 10.1054/jcaf.2002.129263 12555140

[B164] Ross R. S. Borg T. K. (2001). Integrins and the myocardium. Circ. Res. 88, 1112–1119. 10.1161/hh1101.091862 11397776

[B165] Saadat S. Noureddini M. Mahjoubin-Tehran M. Nazemi S. Shojaie L. Aschner M. (2020). Pivotal role of TGF-β/Smad signaling in cardiac fibrosis: non-Coding RNAs as effectual players. Front. Cardiovasc Med. 7, 588347. 10.3389/fcvm.2020.588347 33569393 PMC7868343

[B166] Sano M. Fukuda K. Kodama H. Pan J. Saito M. Matsuzaki J. (2000). Interleukin-6 family of cytokines mediate angiotensin II-induced cardiac hypertrophy in rodent cardiomyocytes. J. Biol. Chem. 275, 29717–29723. 10.1074/jbc.M003128200 10843995

[B167] Sasi A. Romaine A. Erusappan P. M. Melleby A. O. Hasic A. Dahl C. P. (2023). Temporal expression and spatial distribution of the proteoglycan versican during cardiac fibrosis development. Matrix Biol. Plus 19–20, 100135. 10.1016/j.mbplus.2023.100135 PMC10709089 38076279

[B168] Sasse P. Malan D. Fleischmann M. Roell W. Gustafsson E. Bostani T. (2008). Perlecan is critical for heart stability. Cardiovasc Res. 80, 435–444. 10.1093/cvr/cvn225 18694874

[B169] Scheller J. Chalaris A. Schmidt-Arras D. Rose-John S. (2011). The pro- and anti-inflammatory properties of the cytokine interleukin-6. Biochimica Biophysica Acta (BBA) - Mol. Cell Res. 1813, 878–888. 10.1016/j.bbamcr.2011.01.034 21296109

[B170] Schram K. Sweeney G. (2008). Implications of myocardial matrix remodeling by adipokines in obesity-related heart failure. Trends Cardiovasc Med. 18, 199–205. 10.1016/j.tcm.2008.10.001 19185809

[B171] Schultz J. E. J. Witt S. A. Nieman M. L. Reiser P. J. Engle S. J. Zhou M. (1999). Fibroblast growth factor-2 mediates pressure-induced hypertrophic response. J. Clin. Invest 104, 709–719. 10.1172/JCI7315 10491406 PMC408439

[B172] Schumacher S. M. Naga Prasad S. V. (2018). Tumor necrosis Factor-α in heart failure: an updated review. Curr. Cardiol. Rep. 20, 117. 10.1007/s11886-018-1067-7 30259192 PMC6311126

[B173] Schwach V. Passier R. (2019). Native cardiac environment and its impact on engineering cardiac tissue. Biomater. Sci. 7, 3566–3580. 10.1039/c8bm01348a 31338495

[B174] Schwinger R. H. G. Böhm M. Koch A. Schmidt U. Morano I. Eissner H. J. (1994). The failing human heart is unable to use the frank-starling mechanism. Circ. Res. 74, 959–969. 10.1161/01.res.74.5.959 8156643

[B175] Shen J. Z. Morgan J. Tesch G. H. Fuller P. J. Young M. J. (2014). CCL2-dependent macrophage recruitment is critical for mineralocorticoid receptor-mediated cardiac fibrosis, inflammation, and blood pressure responses in male mice. Endocrinology 155, 1057–1066. 10.1210/en.2013-1772 24428529

[B176] Shimojo N. Hashizume R. Kanayama K. Hara M. Suzuki Y. Nishioka T. (2015). Tenascin-C may accelerate cardiac fibrosis by activating macrophages *via* the integrin αVβ3/Nuclear Factor-κB/Interleukin-6 axis. Hypertension 66, 757–766. 10.1161/HYPERTENSIONAHA.115.06004 26238448

[B177] Shirakawa K. Sano M. (2021). Osteopontin in cardiovascular diseases. Biomolecules 11, 1047. 10.3390/biom11071047 34356671 PMC8301767

[B178] Silva A. C. Pereira C. Fonseca A. C. R. G. Pinto-do-Ó P. Nascimento D. S. (2020). Bearing my heart: the role of extracellular matrix on cardiac development, homeostasis, and injury response. Front. Cell Dev. Biol. 8, 621644. 10.3389/fcell.2020.621644 33511134 PMC7835513

[B179] Singh P. Carraher C. Schwarzbauer J. E. (2010). Assembly of Fibronectin extracellular matrix. Annu. Rev. Cell Dev. Biol. 26, 397–419. 10.1146/annurev-cellbio-100109-104020 20690820 PMC3628685

[B180] Spinale F. G. (2007). Myocardial matrix remodeling and the matrix metalloproteinases: influence on cardiac form and function. Physiol. Rev. 87, 1285–1342. 10.1152/physrev.00012.2007 17928585

[B181] Stansfield W. E. Andersen N. M. Tang R. H. Selzman C. H. (2009). Periostin is a novel factor in cardiac remodeling after experimental and clinical unloading of the failing heart. Ann. Thorac. Surg. 88, 1916–1921. 10.1016/j.athoracsur.2009.07.038 19932262 PMC3686640

[B182] Stetler-Stevenson W. G. (2008). Tissue inhibitors of metalloproteinases in cell signaling: metalloproteinase-independent biological activities. Sci. Signal 1, re6. 10.1126/scisignal.127re6 18612141 PMC2493614

[B183] Sun K. yuan Y. Jin J. (2021). A double-edged sword of immuno-microenvironment in cardiac homeostasis and injury repair. Signal Transduct. Target. Ther. 6 (1), 79–16. 10.1038/s41392-020-00455-6 33612829 PMC7897720

[B184] Sweeney M. Corden B. Cook S. A. (2020). Targeting cardiac fibrosis in heart failure with preserved ejection fraction: mirage or miracle? EMBO Mol. Med. 12, e10865. 10.15252/emmm.201910865 32955172 PMC7539225

[B185] Sziksz E. Pap D. Lippai R. Béres N. J. Fekete A. Szabó A. J. (2015). Fibrosis related inflammatory mediators: role of the IL-10 cytokine family. Mediat. Inflamm. 2015, 764641. 10.1155/2015/764641 PMC4495231 26199463

[B186] Tacer K. F. Bookout A. L. Ding X. Kurosu H. John G. B. Wang L. (2010). Research resource: comprehensive expression atlas of the fibroblast growth factor system in adult mouse. Mol. Endocrinol. 24, 2050–2064. 10.1210/me.2010-0142 20667984 PMC2954642

[B187] Takahashi R. Negishi K. Watanabe A. Arai M. Naganuma F. Ohyama Y. (2011). Serum syndecan-4 is a novel biomarker for patients with chronic heart failure. J. Cardiol. 57, 325–332. 10.1016/j.jjcc.2011.01.012 21397460

[B188] Takahashi S. Geenen D. Nieves E. Iwazumi T. (1999). Collagenase degrades collagen *in vivo* in the ischemic heart. Biochimica Biophysica Acta (BBA) - General Subj. 1428, 251–259. 10.1016/s0304-4165(99)00090-2 10434043

[B189] Takawale A. Zhang P. Patel V. B. Wang X. Oudit G. Kassiri Z. (2017). Tissue inhibitor of matrix Metalloproteinase-1 promotes myocardial fibrosis by mediating CD63-Integrin β1 interaction. Hypertension 69, 1092–1103. 10.1161/HYPERTENSIONAHA.117.09045 28373589

[B190] Tallquist M. D. Molkentin J. D. (2017). Redefining the identity of cardiac fibroblasts. Nat. Rev. Cardiol. 14, 484–491. 10.1038/nrcardio.2017.57 28436487 PMC6329009

[B191] Talman A. H. Psaltis P. J. Cameron J. D. Meredith I. T. Seneviratne S. K. Wong D. T. L. (2014). Epicardial adipose tissue: far more than a fat depot. Cardiovasc Diagn Ther. 4, 416–429. 10.3978/j.issn.2223-3652.2014.11.05 25610800 PMC4278038

[B192] Talman V. Ruskoaho H. (2016). Cardiac fibrosis in myocardial infarction—from repair and remodeling to regeneration. Cell Tissue Res. 365 (3), 563–581. 10.1007/s00441-016-2431-9 27324127 PMC5010608

[B193] Teekakirikul P. Eminaga S. Toka O. Alcalai R. Wang L. Wakimoto H. (2010). Cardiac fibrosis in mice with hypertrophic cardiomyopathy is mediated by Non-myocyte proliferation and requires Tgf-β. J. Clin. Invest 120, 3520–3529. 10.1172/JCI42028 20811150 PMC2947222

[B194] Tian M. Yuan Y.-C. Li J.-Y. Gionfriddo M. R. Huang R.-C. (2015). Tumor necrosis factor-α and its role as a mediator in myocardial infarction: a brief review. Chronic Dis. Transl. Med. 1, 18–26. 10.1016/j.cdtm.2015.02.002 29062983 PMC5643772

[B195] Timpl R. Sasaki T. Kostka G. Chu M. L. (2003). Fibulins: a versatile family of extracellular matrix proteins. Nat. Rev. Mol. Cell Biol. 4 (6), 479–489. 10.1038/nrm1130 12778127

[B196] Toba H. Cannon P. L. Yabluchanskiy A. Iyer R. P. D'Armiento J. Lindsey M. L. (2017). Transgenic overexpression of macrophage matrix metalloproteinase-9 exacerbates age-related cardiac hypertrophy, vessel rarefaction, inflammation, and fibrosis. Am. J. Physiol. Heart Circ. Physiol. 312, H375-H383–H383. 10.1152/ajpheart.00633.2016 28011588 PMC5402013

[B197] Travers J. G. Kamal F. A. Robbins J. Yutzey K. E. Burns C. (2017). Cardiac fibrosis: the fibroblast awakens. Circ. Res. 118, 1021–1040. 10.1161/CIRCRESAHA.115.306565 PMC4800485 26987915

[B198] Tucker R. P. Chiquet-Ehrismann R. (2009). The regulation of tenascin expression by tissue microenvironments. Biochim. Biophys. Acta 1793, 888–892. 10.1016/j.bbamcr.2008.12.012 19162090

[B199] Uesugi N. Sakata N. (2005). Role of integrins, including alpha8, for neointima formation after vascular injury. Cardiovasc Res. 65, 766–767. 10.1016/j.cardiores.2005.01.003 15721854

[B200] Valencik M. L. Zhang D. Punske B. Hu P. McDonald J. A. Litwin S. E. (2006). Integrin activation in the heart: a link between electrical and contractile dysfunction? Circ. Res. 99, 1403–1410. 10.1161/01.RES.0000252291.88540.ac 17095723

[B201] Verdecchia P. Angeli F. Mazzotta G. Garofoli M. Ramundo E. Gentile G. (2012). Day-night dip and early-morning surge in blood pressure in hypertension: prognostic implications. Hypertension 60, 34–42. 10.1161/HYPERTENSIONAHA.112.191858 22585951

[B202] Villarreal F. J. Dillmann W. H. (1992). Cardiac hypertrophy-induced changes in mRNA levels for TGF-beta 1, fibronectin, and collagen. Am. J. Physiol. 262, H1861–H1866. 10.1152/ajpheart.1992.262.6.H1861 1535758

[B203] Vistnes M. Aronsen J. M. Lunde I. G. Sjaastad I. Carlson C. R. Christensen G. (2014). Pentosan polysulfate decreases myocardial expression of the extracellular matrix enzyme ADAMTS4 and improves cardiac function *in vivo* in rats subjected to pressure overload by aortic banding. PLoS One 9, e89621. 10.1371/journal.pone.0089621 24595230 PMC3940660

[B204] Waleczek F. J. G. Sansonetti M. Xiao K. Jung M. Mitzka S. Dendorfer A. (2022). Chemical and mechanical activation of resident cardiac macrophages in the living myocardial slice *ex vivo* model. Basic Res. Cardiol. 117, 63–18. 10.1007/s00395-022-00971-2 36449104 PMC9712328

[B205] WalyEldeen A. A. Sabet S. Anis S. E. Stein T. Ibrahim A. M. (2024). FBLN2 is associated with basal cell markers Krt14 and ITGB1 in mouse mammary epithelial cells and has a preferential expression in molecular subtypes of human breast cancer. Breast Cancer Res. Treat. 208, 673–686. 10.1007/S10549-024-07447-Y 39110274 PMC11522194

[B206] Wang J. Hoshijima M. Lam J. Zhou Z. Jokiel A. Dalton N. D. (2006). Cardiomyopathy associated with microcirculation dysfunction in laminin alpha4 chain-deficient mice. J. Biol. Chem. 281, 213–220. 10.1074/jbc.M505061200 16204254

[B207] Wang X. LeMaire S. A. Chen L. Carter S. A. Shen Y. H. Gan Y. (2005). Decreased expression of fibulin-5 correlates with reduced elastin in thoracic aortic dissection. Surgery 138, 352–359. 10.1016/j.surg.2005.06.006 16153447

[B208] Wang X. Lu Y. Xie Y. Shen J. Xiang M. (2019). Emerging roles of proteoglycans in cardiac remodeling. Int. J. Cardiol. 278, 192–198. 10.1016/j.ijcard.2018.11.125 30528626

[B209] Wang Y. Chaffee T. S. LaRue R. S. Huggins D. N. Witschen P. M. Ibrahim A. M. (2020). Tissue-resident macrophages promote extracellular matrix homeostasis in the mammary gland stroma of nulliparous mice. Elife 9, 1–27. 10.7554/eLife.57438 PMC7297528 32479261

[B210] Weber S. Saftig P. (2012). Ectodomain shedding and ADAMs in development. Development 139, 3693–3709. 10.1242/dev.076398 22991436

[B211] Widyantoro B. Emoto N. Nakayama K. Anggrahini D. W. Adiarto S. Iwasa N. (2010). Endothelial cell-derived endothelin-1 promotes cardiac fibrosis in diabetic hearts through stimulation of endothelial-to-mesenchymal transition. Circulation 121, 2407–2418. 10.1161/CIRCULATIONAHA.110.938217 20497976

[B212] Wigle E. D. Sole M. J. Williams W. C. Williams W. C. Rojkind M. (1991). Pathologic fibrosis and matrix connective tissue in the subaortic myocardium of patients with hypertrophic cardiomyopathy. J. Am. Coll. Cardiol. 17, 1343–1351. 10.1016/s0735-1097(10)80145-7 2016452

[B213] Xu S. Zhang J. Liu J. Ye J. Xu Y. Wang Z. (2021). The role of interleukin-10 family members in cardiovascular diseases. Int. Immunopharmacol. 94, 107475. 10.1016/j.intimp.2021.107475 33662690

[B214] Yabluchanskiy A. Ma Y. Iyer R. P. Hall M. E. Lindsey M. L. (2013). Matrix Metalloproteinase-9: many shades of function in cardiovascular disease. Physiology 28, 391–403. 10.1152/physiol.00029.2013 24186934 PMC3858212

[B215] Yan W. Wang P. Zhao C. X. Tang J. Xiao X. Wang D. W. (2009). Decorin gene delivery inhibits cardiac fibrosis in spontaneously hypertensive rats by modulation of transforming growth factor-beta/smad and p38 mitogen-activated protein kinase signaling pathways. Hum. Gene Ther. 20, 1190–1200. 10.1089/hum.2008.204 19697998

[B216] Yang Y. Yu W. Yan W. Xia Q. (2021). Decorin induces cardiac hypertrophy by regulating the CaMKII/MEF-2 signaling pathway *in vivo* . Curr. Med. Sci. 41, 857–862. 10.1007/s11596-021-2426-y 34643879

[B217] Ye J. Wang Z. Ye D. Wang Y. Wang M. Ji Q. (2019). Increased Interleukin-11 levels are correlated with cardiac events in patients with chronic heart failure. Mediat. Inflamm. 2019, 1575410. 10.1155/2019/1575410 PMC6341241 30728748

[B218] Yildirim Z. Swanson K. Wu X. Zou J. Wu J. (2025). Next-gen therapeutics: pioneering drug discovery with iPSCs, genomics, AI, and clinical trials in a dish. Annu. Rev. Pharmacol. Toxicol. 65, 71–90. 10.1146/annurev-pharmtox-022724-095035 39284102 PMC12011342

[B219] Yokota T. McCourt J. Ma F. Ren S. Li S. Kim T. H. (2020). Type V collagen in scar tissue regulates the size of scar after heart injury. Cell 182, 545–562. 10.1016/j.cell.2020.06.030 32621799 PMC7415659

[B220] Yokoyama T. Nakano M. Bednarczyk J. L. McIntyre B. W. Entman M. Mann D. L. (1997). Tumor necrosis Factor-α provokes a hypertrophic growth response in adult cardiac myocytes. Circulation 95, 1247–1252. 10.1161/01.cir.95.5.1247 9054856

[B221] Yousif L. F. Di Russo J. Sorokin L. (2013). Laminin isoforms in endothelial and perivascular basement membranes. Cell Adh Migr. 7, 101–110. 10.4161/cam.22680 23263631 PMC3544773

[B222] Zhang H. Wu J. Dong H. Khan S. A. Chu M. L. Tsuda T. (2014). Fibulin-2 deficiency attenuates angiotensin II-induced cardiac hypertrophy by reducing transforming growth factor-β signalling. Clin. Sci. (Lond) 126, 275–288. 10.1042/CS20120636 23841699 PMC4075193

[B223] Zhao S. Wu H. Xia W. Chen X. Zhu S. Zhang S. (2014). Periostin expression is upregulated and associated with myocardial fibrosis in human failing hearts. J. Cardiol. 63, 373–378. 10.1016/j.jjcc.2013.09.013 24219836

[B224] Zibadi S. Cordova F. Slack E. H. Watson R. R. Larson D. F. (2011). Leptin’s regulation of obesity-induced cardiac extracellular matrix remodeling. Cardiovasc Toxicol. 11, 325–333. 10.1007/s12012-011-9124-0 21744298

